# Imbalance-Aware Machine Learning for Predicting Rare and Common Disease-Associated Non-Coding Variants

**DOI:** 10.1038/s41598-017-03011-5

**Published:** 2017-06-07

**Authors:** Max Schubach, Matteo Re, Peter N. Robinson, Giorgio Valentini

**Affiliations:** 1 Institute for Medical and Human Genetics, Charité – Universitätsmedizin Berlin, Augustenburger Platz 1, 13353 Berlin, Germany; 2 Anacletolab, Dipartimento di Informatica, Università degli Studi di Milano, Via Comelico 39, 20135 Milan, Italy; 3 The Jackson Laboratory for Genomic Medicine, 10 Discovery Drive, Farmington, CT 06032 USA; 4 Institute for Systems Genomics, University of Connecticut, Farmington, CT 06032 USA

## Abstract

Disease and trait-associated variants represent a tiny minority of all known genetic variation, and therefore there is necessarily an imbalance between the small set of available disease-associated and the much larger set of non-deleterious genomic variation, especially in non-coding regulatory regions of human genome. Machine Learning (ML) methods for predicting disease-associated non-coding variants are faced with a chicken and egg problem - such variants cannot be easily found without ML, but ML cannot begin to be effective until a sufficient number of instances have been found. Most of state-of-the-art ML-based methods do not adopt specific imbalance-aware learning techniques to deal with imbalanced data that naturally arise in several genome-wide variant scoring problems, thus resulting in a significant reduction of sensitivity and precision. We present a novel method that adopts imbalance-aware learning strategies based on resampling techniques and a hyper-ensemble approach that outperforms state-of-the-art methods in two different contexts: the prediction of non-coding variants associated with Mendelian and with complex diseases. We show that imbalance-aware ML is a key issue for the design of robust and accurate prediction algorithms and we provide a method and an easy-to-use software tool that can be effectively applied to this challenging prediction task.

## Introduction

Next Generation Sequencing (NGS) and especially whole-genome sequencing (WGS) is rapidly and profoundly transforming medical genetics because of its ability to investigate genomic variation in coding and in non-coding regions across the entire human genome^1–3^. Especially the detection of rare and common disease-associated non-coding variants is a challenging problem and recently several machine learning (ML) methods have been proposed to identify or prioritize “relevant” non-coding genetic variants^4–9^. Despite their successful application to several problems in medical genetics, a limitation of these approaches is that they require a significant number of “positive” instances (e.g. variants known to be associated with a specific disease) to generalize and correctly predict novel associations. However, disease and trait-associated variants represent only a tiny minority of all known genetic variation. For instance in non-coding regions the number of available positive examples for Mendelian diseases is of the order of several hundreds, while the number of negative examples is of the order of millions^10^. Similar conditions, with different levels of imbalance between positive and negative examples, may arise in other medically relevant contexts, e.g. in variants related to cancer^11^, or in Genome Wide Association Studies (GWAS) for complex diseases^12^.

The vast majority of trait- or complex-disease-associated variants identified to date in GWAS have been found to be located outside of protein-coding sequences and in some cases localize to known gene regulatory elements such as promoters and enhancers. It should be noted though that SNPs on genotyping chips are “tags” for haplotypes on which functional variants reside, rather than necessarily being disease-causing themselves^13, 14^. Relatively few non-coding variants causal for Mendelian disease have been identified to date. Although historically variation in non-coding sequences has remained underinvestigated^15^, mutations have been confidently identified in a wide range of non-coding functional elements, including promoters, enhancers, 5′ and 3′ UTRs, RNA genes, and imprinting control elements^10^. A better understanding of regulatory variants will therefore be necessary to unravel the functional architecture of rare and common disease.

However, computational algorithms for the analysis of non-coding deleterious variants are faced with special challenges owing to the rarity of confirmed pathogenic mutations. In this setting, classical learning algorithms, such as support vector machines^16^ or artificial neural networks^17^ tend to generalize poorly, because they usually predict the minority class with very low sensitivity and precision^18^. In the context of the prediction of genetic variants associated with traits or diseases, this boils down to wrongly predicting most of the disease-associated variants as non-disease associated, thus significantly limiting the usefulness of ML methods for the prediction of novel, disease-associated non-coding variants.

To address this problem we have developed *hyperSMURF*, hyper-ensemble of SMOTE Undersampled Random Forests, a method specifically designed for the analysis of imbalanced genomic data. *HyperSMURF* adopts imbalance-aware learning strategies based on resampling techniques and a hyper-ensemble approach: the simultaneous oversampling of the minority class and undersampling of the majority class generates balanced training sets, each one used to train a different random forest ensemble. The predictions of the trained models are finally combined through an hyper-ensemble approach (ensemble of ensembles) to obtain an overall “consensus” prediction. The burden of the computation can be reduced by using parallel computation techniques, since the learning processes of the random forest ensembles are largely independent and easily parallellizable. Moreover the diversity between the learners and the balancing between positive and negative examples introduced by the over and undersampling techniques avoid the bias toward the majority class and promote good generalization behavior, while the hyper-ensemble approach provides more accurate base learners and robust predictions.

To show the effectiveness of the proposed approach, we have performed an experimental comparison with state-of-the-art ML methods for two challenging and medically relevant ML prediction problems for imbalanced genomic data: the prediction of regulatory mutations underlying Mendelian diseases^10^ and the prediction of non-coding variants associated with GWAS regulatory hits from the GWAS-Catalog^19^. For the first time, to our knowledge, we retrained and tested different state-of-the-art methods on the same data and genomic features instead of using pre-trained models or pre-computed scores as it has been usually done in previous work^4–6, 9, 10^. Results show that imbalance-aware ML strategies can successfully counteract the bias toward the majority class of state-of-the-art ML methods, and are essential for predicting disease-associated non-coding variants in genomic contexts characterized by a large imbalance between the known small set of available rare and common disease-associated variants and the huge set of all known human genetic variation.

## Methods

**Figure 1:**
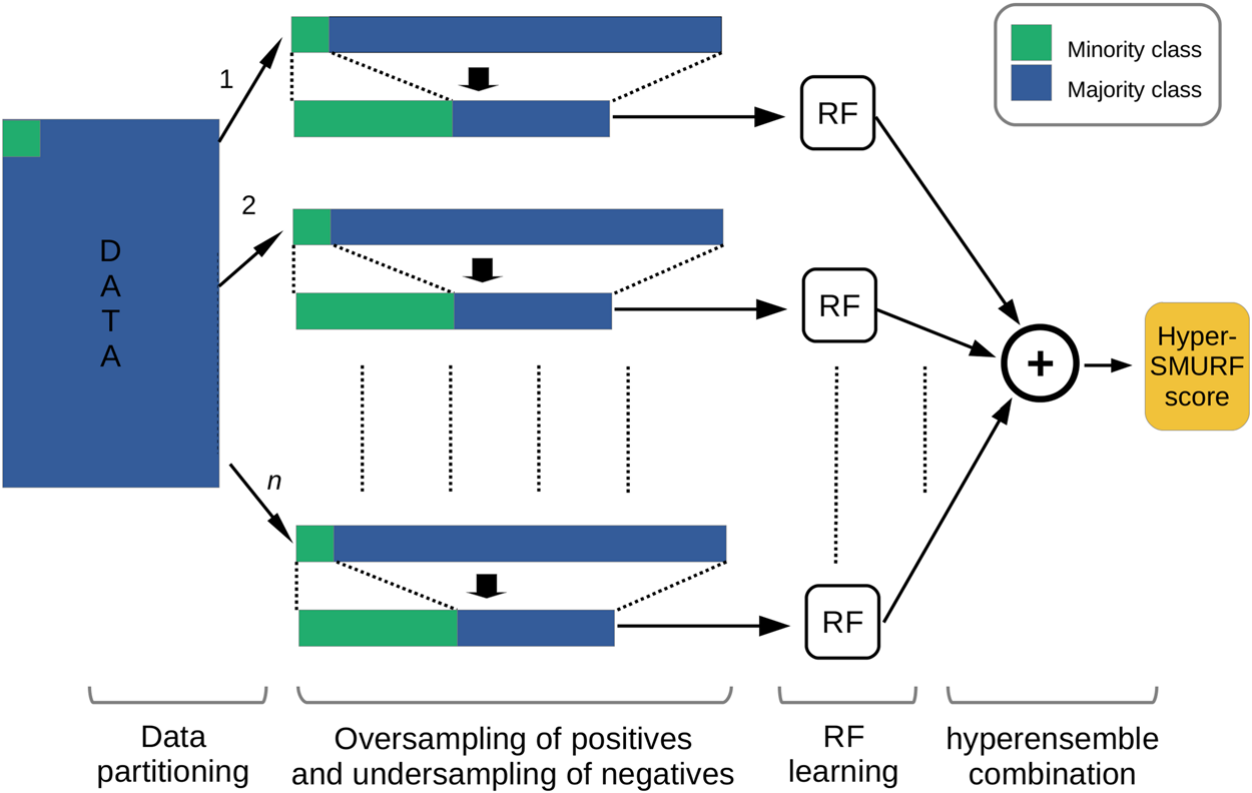
A schematic representation of the *hyperSMURF* method. *HyperSMURF* divides the majority class (the negative class of probably non-deleterious variants–blue rectangles) into *n* partitions. For each partition, oversampling techniques are used to generate additional examples from the minority class (the positive class of deleterious variants–green rectangles), that closely resemble the distribution of the actual positive examples within the vector space of genomic attributes, to amplify the number of training examples from the minority class. At the same time a comparable number of examples is subsampled from the majority class. Then *hyperSMURF* trains in parallel *n* random forests on the resulting balanced data sets and finally combines the prediction of the *n* ensembles according to a hyper-ensemble (ensemble of ensembles) approach.

*HyperSMURF* is a method specifically conceived to deal with imbalanced data for the prediction of deleterious (e.g. disease-associated) variants in the human genome. To address this challenge and to achieve high coverage of the available input data as well as a high accuracy of the predictions, *hyperSMURF* is based on three complementary strategies: 1) Sampling techniques; 2) Ensembling methods; 3) Hyper-ensemble approach (Fig. 1).

### Sampling techniques

**Figure 2:**
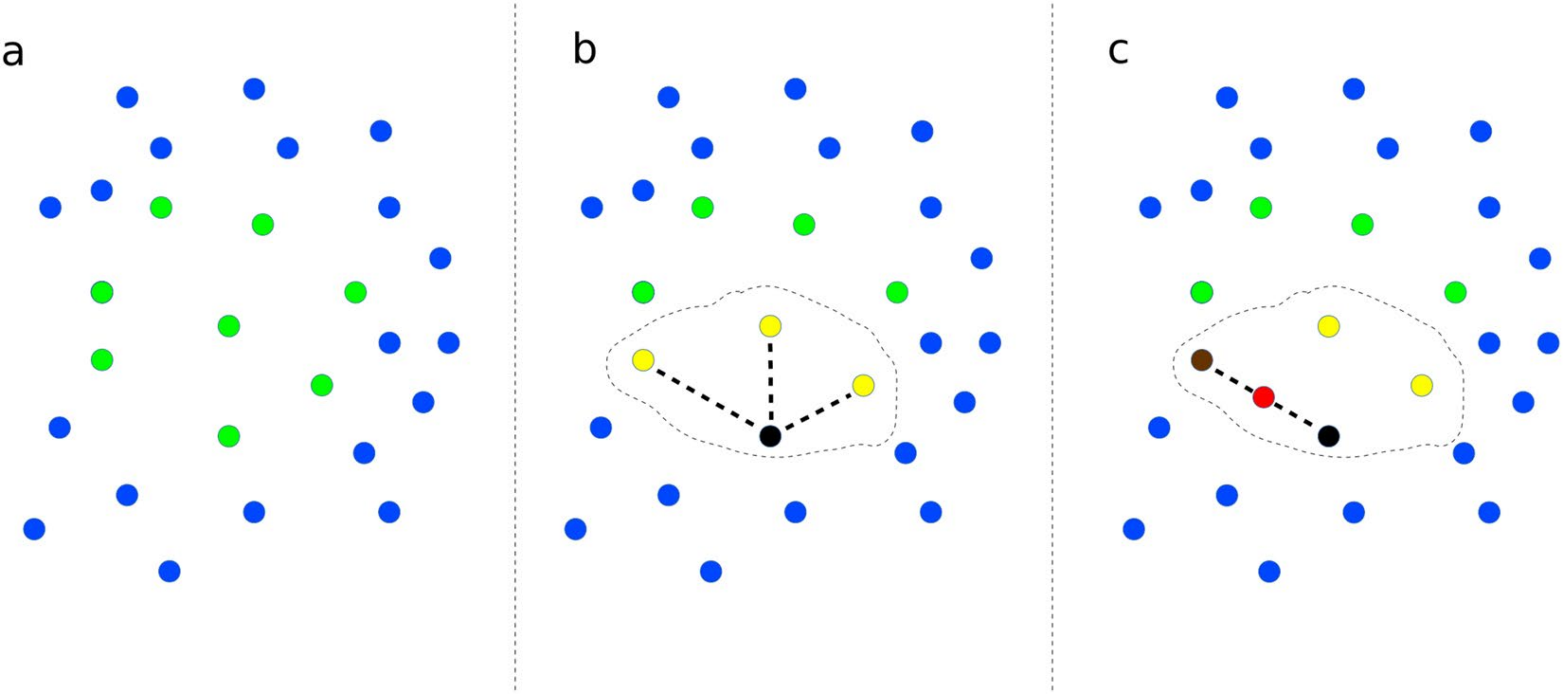
Graphical representation of the *SMOTE* algorithm. (**a**) *SMOTE* starts from a set of positive (green points) and negative (blue points) examples; (**b**) It then selects a positive example (black) and its *k* nearest neighbors among the positives (yellow points, with *k* = 3), (**c**) Finally one of the *k* nearest neighbours is randomly selected (brown point) and a new synthetic positive example is added, by randomly generating an example (red point) along the straight line that connects the black and brown points. The procedure depicted in (**b**,**c**) is repeated for all the positives, by adding each time a new synthetic example similar (in an Euclidean sense) to the other positive examples.

The majority class, composed of non-deleterious variants (negative examples), is subdivided into *n* non-overlapping partitions. Then non-deleterious variants are randomly subsampled to reduce the number of negative examples in each partition. At the same time oversampling techniques are applied to the minority class, composed by deleterious variants (positive examples) to enlarge their number. For oversampling, we used the synthetic minority over-sampling technique (SMOTE)^20^, which generates new synthetic positive examples similar to the original data (see Fig. 2 for more details). Finally, for each subsampled partition of non-deleterious variants a different oversampled set of deleterious variants is added, resulting in balanced data sets (i.e. data sets with a comparable number of positive and negative examples).

### Ensembling methods

Each balanced data set *d* does not in itself assure a sufficient coverage of the available training data, since only a small subset of the negative training data is included in each data set *d*. To overcome this limitation, a different learning machine is trained on each of the *n* different data sets *d* and then the predictions of the *n* resulting models are combined according to ensembling techniques to obtain a “consensus” prediction of the ensemble. Note that each base learner is trained on different training data, thus assuring diversity between the base learners, a key concept for the success of ensemble methods^21^. At the same time a high coverage is guaranteed, since the *n* data sets include a significant part of the available training data.

### Hyper-ensemble approach

Another key factor for the success of ensemble methods is the accuracy of the base learners^22^, and it is well-known that ensembles usually improve the accuracy and the robustness of the predictions of the learning machines^23, 24^. To this end, we trained *n* random forests (RFs)^25^, i.e. ensembles of decision trees, one for each data set *d*, and then combined their predictions by averaging across the probabilities estimated by each RF, thus resulting in a hyper-ensemble (an ensemble of ensembles), because each base learner is in turn an ensemble of decision trees. In principle any ensemble of learning machines can be used but we have chosen RFs because they have been shown to be effective for the analysis of genetic variants^5, 26^.

### Pseudocode and details of the algorithm

**Figure 3:**
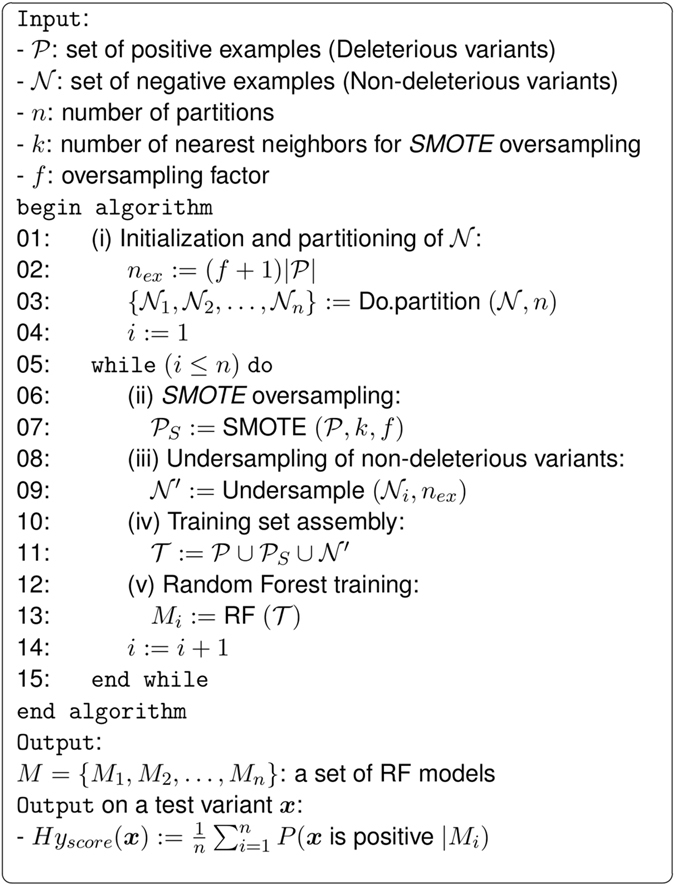
The *hyperSMURF* algorithm.

Figure 3 shows the pseudocode of the *hyperSMURF* algorithm. ${\mathscr{P}}$ represents the set of positive examples, i.e. the set of available deleterious variants, while ${\mathscr{N}}$ is the set of negative examples, i.e. the non-deleterious non-coding variants that we assume to greatly outnumber ${\mathscr{P}}$. More precisely ${\mathscr{P}}=\{({\boldsymbol{x}},y)|{\boldsymbol{x}}\in F,y=1\}$ and ${\mathscr{N}}=\{({\boldsymbol{x}},y)|{\boldsymbol{x}}\in F,y=0\}$, where *y* ∈ {0, 1} represents the two different classes of deleterious (*y* = 1) and non-deleterious variants (*y* = 0), and *F* is a set of real-valued vectors representing the features associated with each variant.

At first the algorithm initializes some parameters, such as *n*_*ex*_, the number of positive and the number of negative examples to be used to train each base learner (line 2). Note that $|{\mathscr{P}}|$ represents the cardinality of the set ${\mathscr{P}}$ and *f* ≥ 1 the oversampling factor, i.e., how much we would like to oversample the minority class of the positive examples. Then the algorithm subdivides the negative examples ${\mathscr{N}}$ in *n* partitions $\{{{\mathscr{N}}}_{1},{{\mathscr{N}}}_{2},\ldots ,{{\mathscr{N}}}_{n}\}$, such that ${\cup }_{i=1}^{n}{{\mathscr{N}}}_{i}={\mathscr{N}}$ and ${\cap }_{i=1}^{n}{{\mathscr{N}}}_{i}=\varnothing$ (line 3).

The while loop (lines 5–15) iterates a set of steps on the *n* partitions of the data to train a different RF at each iteration^25^. In the first step, a set of synthetic examples ${{\mathscr{P}}}_{S}$ is generated from the set of available positive examples ${\mathscr{P}}$ through the Synthetic Minority Over-sampling Technique^20^ (*SMOTE*, line 7). This algorithm generates “realistic” novel examples that are similar to the original examples by linear interpolation between randomly chosen pairs of close positive examples. More precisely for each ${\boldsymbol{x}}\in {\mathscr{P}}$ the first *k* positive nearest neighbors $KNN\subset {\mathscr{P}}$ are computed and an example ***x***′ ∈ *KNN* is randomly drawn. Then a new synthetic example ***x***_*s*_ is computed by linear interpolation between them:

1 $${{\boldsymbol{x}}}_{s}={\boldsymbol{x}}+\lambda ({\boldsymbol{x}}^{\prime} -{\boldsymbol{x}})$$

where *λ* is a random number between 0 and 1. This process is repeated *f* times for each example ${\boldsymbol{x}}\in {\mathscr{P}}$ and hence the resulting “synthetic” data set ${{\mathscr{P}}}_{S}$ has a cardinality $|{{\mathscr{P}}}_{S}|=f|{\mathscr{P}}|$ (see Fig. 2 for an intuitive geometric representation of the *SMOTE* algorithm).

Then a subsample of negative examples ${\mathscr{N}}\,^{\prime}$ is constructed by randomly subsampling *n*_*ex*_ examples from the partition ${{\mathscr{N}}}_{i}$ in order to obtain $|{\mathscr{N}}\,^{\prime} |=|{\mathscr{P}}|+|{{\mathscr{P}}}_{S}|$, that is a number of negative examples equal to that of positives (line 9). The training set ${\mathscr{T}}$ is obtained by simple union of all positives, the sampled positives and negative examples (line 11). At each iteration of the while loop the resulting training set ${\mathscr{T}}$ is used to train a RF model *M*_*i*_ (line 13) which is able to compute the probability *P*(***x*** is positive|*M*_*i*_) that a given variant ***x*** is deleterious (e.g. associated with a genetic disease). The output of the algorithm is a set *M* of RFs. Finally the *hyperSMURF* score (*Hy*_*score*_) for a given variant ***x*** is computed by averaging across the probabilities estimated by each different base RF:

2 $$H{y}_{score}({\boldsymbol{x}}):=\frac{1}{n}\sum _{i=1}^{n}\,P({\boldsymbol{x}}\,{\rm{is}}\,{\rm{positive}}|{M}_{i})$$

In our setting each base learner *M*_*i*_ is a RF, which is in turn an ensemble of decision trees, thus resulting in an ensemble of ensembles (hyper-ensemble). By changing line 2 of the algorithm to ${n}_{ex}:=m(f+1)|{\mathscr{P}}|$ where *m* ≥ 1, we can obtain $|{\mathscr{N}}\,^{\prime} |=m(|{\mathscr{P}}|+|{{\mathscr{P}}}_{S}|)$, i.e. negative samples ${\mathscr{N}}^{\prime}$ whose cardinality is *m* times that of the positive samples: in this way by enlarging the undersampling factor *m* we can exploit a larger set of negative examples for training. For instance in our experiments we used *m* = 3 to better exploit the available negative examples.

In sum, by adopting both over- and undersampling techniques^20, 27, 28^ to increase the count of the positive examples and reduce the cardinality of the negatives, *hyperSMURF* obtains balanced data sets for training the RFs. Moreover the ensemble approach assures a high coverage of the available training data with diverse base learners, since each RF is trained on different training data. Finally the hyper-ensemble approach uses ensembles as base learners, instead of single learning machines, which assures accurate and robust predictions.

It is worth noting that we recently proposed *ReMM* a related but different algorithm as part of the *Genomiser* method^10^. *HyperSMURF*, by tuning its learning parameters (*n*, *f*, *m*, *k*, see above) can be adapted to different score ranking problems, while *ReMM* is a non-parametric method tailored to Mendelian diseases only. Moreover *ReMM* is not a stand-alone method, but is part of *Genomiser*, a computational approach tailored to the discovery of pathogenic variants related to specific Mendelian diseases, where phenotypic information is considered too, while *hyperSMURF* is a stand-alone method for scoring potentially deleterious variants related to “classes” of diseases (e.g. variants associated to rare or common diseases), not to a specific disease and it uses only genomic information for its predictions.

## Data and experimental set-up

We considered two case studies for the prediction of deleterious variants in non-coding regions: a) Mendelian diseases b) complex diseases. Both case studies are characterized by imbalanced data sets and different sets of features associated with each variant. The first data set includes about 400 manually curated regulatory mutations associated with Mendelian diseases (positive examples) and about 14 million presumedly non-deleterious variants in non-coding regions (negative examples) annotated with 26 genomic attributes extracted from different sources^10^, and the second one about 2000 GWAS regulatory hits from the GWAS-Catalog^19^ and about 1.4 million non-deleterious variants, annotated with about 1800 genomic attributes directly extracted from the DNA sequence through deep convolutional networks^6^. We observe that in principle also with Mendelian diseases we could use genomic attributes similar to those used with GWAS regulatory hits, but we intentionally considered two different prediction problems in non-coding regions to show the effectiveness of imbalance-aware ML methods in two cases characterized by very different low dimensional (Mendelian) and high dimensional (GWAS) genomic features contexts.

### Mendelian data

The data set contains regulatory Mendelian mutations in non-coding regions of the human genome and its detailed description is available from ref. 10. The positive set includes 406 manually curated mutations located in genome regions such as 5′ and 3′ UTR, enhancers, and promoters. Their pathogenic contribution to a Mendelian disease is highly reliable because of comprehensive biomedical literature review whereby only variants with strong evidence supporting their pathogenicity were selected (e.g. because of familial cosegregation or experimental validation)^10^.

Creation of negative (non-deleterious) variants was similar to the approach of CADD^4^. Single-nucleotides were identified in the human reference genome sequence (hg19) differing, in high confidence alignment regions, from the ancestral primate genome sequence inferred on the basis of the Ensembl Enredo-Pechan-Ortheus (EPO) whole-genome alignments of six primate species^29, 30^ (Ensembl Compara release e71). In order to filter out variants that had not been exposed to many generations of natural selection all identified single-nuclotides that were present in the most recent 1000 Genomes data^31^ with frequency higher than 5% were removed. Finally, to gain non-coding negatives, positions in the protein coding regions were removed according to the content of the NCBI Reference Sequence (RefSeq) database (annotation release 105). This led to the definition of a negative set ${{\mathscr{N}}}_{M}$ containing 14,755,199 variants (see Supplementary Table 1 for the distribution of variants to functional categories in ${{\mathscr{N}}}_{M}$).

The different amounts of positives and negatives results in an extreme imbalance of ~1:36,000, i.e. one positive regulatory Mendelian mutation every 36,000 negative non-deleterious variants. The genomic attributes associated to each variant have been obtained from different sources, including conservation scores, transcription and regulation annotations, G/C content, population-based features and others, thus resulting in a vector of 26 genomic features that were used for training (see Supplementary Table 2 for details).

### GWAS data

We used 2115 regulatory GWAS hits downloaded on May 13, 2016 from https://xioniti01.u.hpc.mssm.edu/v1.0/EIGEN_TestDatasets/GWAS_eQTLs/GWAScatalog_EIGEN.txt ^9^ (date of access: 03/07/2017). Regulatory hits were defined by the authors as GWAS hits that overlap with a known regulatory element, according to the National Human Genome Research Institute (NHGRI) GWAS catalog and these hits were also the variations that received the highest Eigen scores^9^. We used the same non-deleterious sites as the negative common-disease training set as for the Mendelian data, but we removed every position overlapping with the regulatory GWAS hits.

GWAS hits and negative variants were converted into VCF format. More precisely DeepSEA^6^ was used to extract their chromatin effect features. For each of the aforementioned variants we performed a DeepSEA analysis (DeepSEA version: 0.94, genome assembly: GRCh37/hg19, code for the standalone version and additional evolutionary conservation score files downloaded from http://deepsea.princeton.edu/media/code/deepsea.v0.94.tar.gz (date of access: 03/07/2017). in order to produce a set of genomic attributes directly extracted from the DNA sequence through deep convolutional networks.

Using the DeepSEA feature extraction the feature set is larger than the one used in the Mendelian data. To this end we parsed the intermediate files produced for each variant by DeepSEA. The deep convolutional network at the core of the DeepSEA method makes predictions for 919 chromatin features (125 DNase features, 690 Transcription Factor features, 104 histone features). For each of the considered variants DeepSEA predicts two score values by analyzing two 1000-bp sequences centered on the variant based on the reference genome and carrying the reference and the alternative allele at the variant position (500^*th*^ nucleotide).

We used the predicted chromatin effect scores provided by DeepSEA to compute two set of features: 919 absolute differences of probabilities (*diff*) and 919 relative log fold changes of odds (*log* *fold*), obtaining an initial set of 1838 features:

3 $$\begin{array}{rcl}\quad \,\,diff & = & |P(reference)-P(alternative)|\\ log\,fold & = & |log\frac{P(reference)}{1-P(reference)}-log\frac{P(alternative)}{1-P(alternative)}|\end{array}$$

As shown in Supplementary Fig. 10, *diff* and *logfold* capture distinct features of the difference between *P*(*reference*) and *P*(*alternative*): *diff* represents this difference as the absolute value of a linear function, while *logfold* emphasizes large differences between *P*(*reference*) and *P*(*alternative*).

In addition we extracted from the DeepSEA intermediate output the four precomputed evolutionary scores used (base-level PhastCons score for primates (excluding human), PhyloP score for primates (excluding human), and GERP++ neutral evolution and rejected substitution scores) and finally we obtained a set of 1842 features for each variant. According to the protocol detailed by Zhou *et al*.^6^ all extracted features were normalized to zero mean and unit variance prior to further analysis.

The processing of the features with deep convolutional networks, using an NVIDIA K20 device for GPU parallel computing and a server with 128 GB of RAM required about 115.6 hours of computation time for processing 1,475,911 variants. To reduce the computation time, while maintaining a sufficiently large set of negative background variants, we randomly took only 10% of the negative data (1,475,505 variants in total) resulting in an imbalance of ~1:700.

#### Experimental set-up

Cross-validation (CV) strategies specifically designed for genomic data were used for training and testing to guarantee an unbiased performance evaluation. To compare the performance of *hyperSMURF* we retrained other state-of-the-art methods for variant relevance prediction on our Mendelian and GWAS data.

### Performance evaluation

For model performance testing we ensure that variants of the same location, gene, or disease do not occur jointly in the training and test set and thereby biasing results. To this end a “cytogenetic band-aware” 10-fold CV was made to partition the variants into chromosomal bands. Bands with at least one positive mutation were assigned to one of the ten folds so that each fold contains a similar number of positives. The remaining bands were randomly assigned to different partitions. Negative variants were added to the partition of their associated band. We used different performance measurements, mainly the Area Under the Precision and Recall curve (AUPRC), the Area Under the Receiver Operating Characteristic curve (AUROC), and the precision, recall and F-score as a function of the score threshold.

### Comparison with state-of-the-art methods and software implementation

We compared the imbalance-aware *hyperSMURF* method with state-of-the-art methods for scoring variants: *CADD* ^4^, *GWAVA* ^5^, *DeepSEA* ^6^, and *Eigen* ^9^. Apart from *GWAVA*, which uses a modified version of the RF algorithm by balancing the training data through undersampling of the background variants (the majority class), the other state-of-the-art methods are imbalance-unaware in the sense that they do not adopt specific learning techniques to deal with highly imbalanced data. We point out that we retrained and tested the models using the above data sets instead of using pre-trained models or pre-computed scores as it is often done in previous work^4–6, 9^. To our knowledge this is the first time that such experimental comparison between different state-of-the-art scoring methods has been performed.

*HyperSMURF* was implemented both in Java and R, and the software is publicly available from Maven (Java) and CRAN (R) repositories. The Java implementation is published under GNU GPLv3 and source code is available via GitHub https://github.com/charite/hyperSMURF (date of access: 03/07/2017). For installation details see the website https://charite.github.io/hyperSMURF (hyperSMURF, date of access: 03/07/2017). Version 0.2 of this implementations was used to produce the results. The R version of *hyperSMURF* is available from the CRAN repository: https://cran.r-project.org/package=hyperSMURF (hyperSMURF: Hyper-Ensemble Smote Undersampled Random Forests, date of access: 03/07/2017). Supplementary Note 3 provides a tutorial for the usage of the *hyperSMURF* R package and its application to the analysis of genetic variants.

The Support Vector Machine underlying the *CADD* score was reimplemented by our in-house efficient C++ implementation using the LibLinear library^32^. *GWAVA* was reimplemented using Java and the Weka library^33^. This weka module is named weka-GWAVA (version v0.1) and source code is available from https://charite.github.io/weka-GWAVA (Weka-gwava, date of access: 03/07/2017). Both *CADD* and *GWAVA* methods were reimplemented for improved computational efficiency. The R code for the *Eigen* and *Eigen*-*PC* methods was downloaded from the Eigen Website: http://www.columbia.edu/~ii2135/Eigen_functions_112015.R (date of access: 03/07/2017), while the original *DeepSEA* software was downloaded from http://deepsea.princeton.edu/media/code/deepsea.v0.94.tar.gz (date of access: 03/07/2017).

## Results

In this section we present the results of the experimental comparison between *hyperSMURF* and state-of-the-art scoring methods for the prediction of genetic variants associated with traits or diseases according to the experimental set-up described in the previous Section. First we compared the scoring methods using different metrics and the cytoband-aware 10-fold CV previously described in the Section “Performance evaluation”. Then, to investigate the effect of imbalance in the functional prioritization of rare variants, we performed testing with progressively imbalanced data sets. Finally, we check the capability of *hyperSMURF* to detect disease variants that are close to non-deleterious variants.

### *HyperSMURF* significantly outperforms state-of-the-art methods with imbalanced data

Independently of the metrics used to compare *hyperSMURF* with state-of-the-art scoring methods (AUPRC, AUROC, precision, recall and F-measure at different score threshold levels, analysis of the distribution of top-ranked variants associated with traits or diseases), the results show that *hyperSMURF* achieves significantly better results than the other methods, as detailed in the following sections.

#### Area Under the Precision-Recall Curve (AUPRC) results

**Figure 4:**
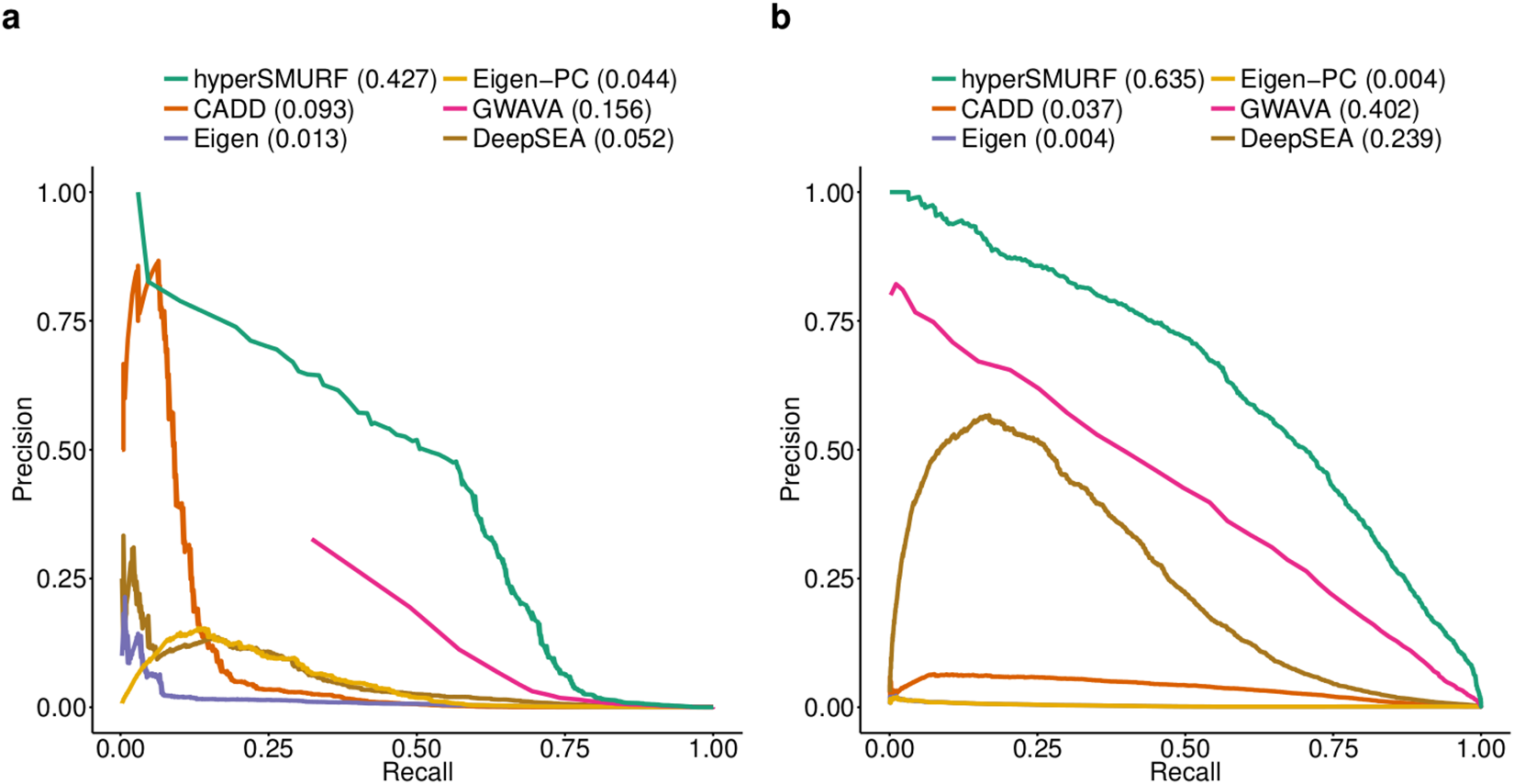
Comparison of the precision/recall curves (PRC) across methods. (**a**) PRC with Mendelian regulatory mutations. (**b**) PRC with GWAS regulatory hits. Numbers in parentheses represent AUPRC values.

**Figure 5:**
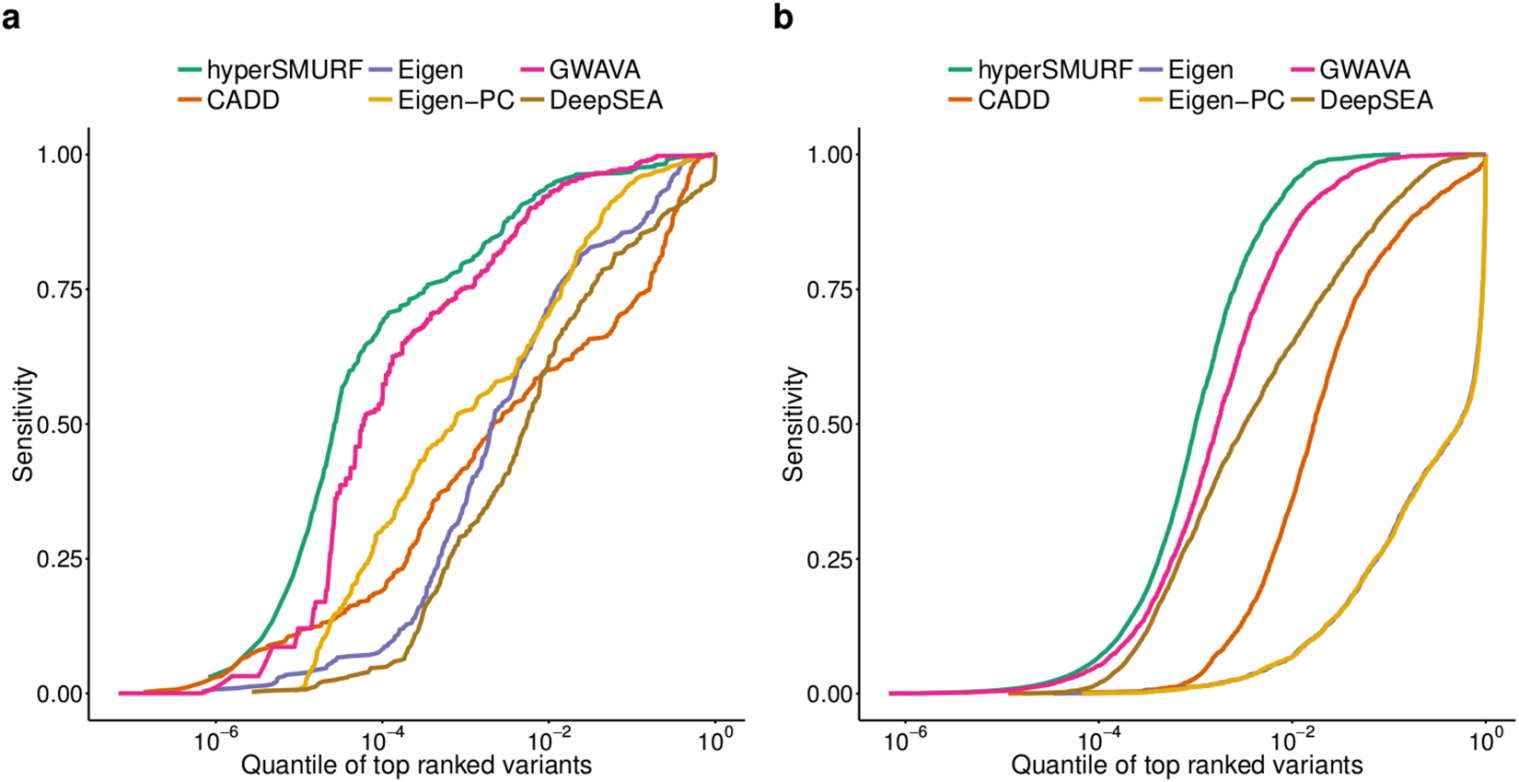
Comparison across methods of the sensitivity with respect to the quantiles of top ranked variants. (**a**) sensitivity with respect to the quantiles of top ranked variants with Mendelian data; (**b**) sensitivity with respect to the quantiles of top ranked variants with the regulatory GWAS hits. X axis is log_10_ transformed.

The overall results in terms of AUPRC show that *hyperSMURF* achieves significantly better results than state-of-the-art methods with both the Mendelian and GWAS data (Fig. 4). At all recall levels *hyperSMURF* (green curve) achieves larger values of precision, and its AUPRC is significantly larger than those the other compared state-of-the-art methods (Fig. 4). These results are confirmed also by the analysis of the ROC curves and of the sensitivity of the methods as a function of the quantiles of top ranked variants (Supplementary Fig. 1 and Fig. 5). Although the differences between the methods as measured by the AUROC are not very large (even though in several cases the differences are statistically significant according to the DeLong test^34^), it is well-known that with imbalanced data the AUPRC is more informative than the AUROC^35, 36^.

The most informative genomic features, according to the average of a 100 times repeated univariate analysis performed through a classical logistic regression model, are represented in both datasets by conservation scores such as PhastCons and their associated -log p-values (PhyloP)^37^ (see Supplementary Tables 6 and 7 for more details). Chromatin effect features are overrepresented in the GWAS data and our univariate analysis shows that every feature is not informative on its own (Supplementary Table 7). Nevertheless we observe that such features have a low coverage over the whole genome, and the information gain introduced by their usage is likely due by their combination. The good performance of *hyperSMURF* on GWAS data shows that the ensembles of RFs are able to meaningfully combine the chromatin features and at the same to exploit the high informativeness of conservation scores.

#### Distribution of top ranked relevant variants associated with traits or diseases

The curves in Fig. 5 represent the sensitivity as a function of the quantile of top ranked “positive” variants. For instance a sensitivity equal to 0.1 at a quantile 10^−3^ means that one tenth of the positive variants lie in the top 0.001 quantile, i.e. in the first top ranked thousandth of the overall variants. As an example, in Fig. 5a the *hyperSMURF* curve shows a sensitivity equal to about 0.75 at a quantile 10^−4^; this means that about 3/4 of the Mendelian mutations in non-coding regions are scored in the top ten thousandth of the overall variants, i.e. about 300 Mendelian mutations are within the top scored 1400 of the about 14 million genetic variants analyzed. In other words, these curves reflect the ability of the different methods to score the “positive” variants in the top positions of the ranking. From this standpoint the curves show that imbalance-aware methods achieve the best results, since *hyperSMURF* curves (green) and *GWAVA* curves (pink) lie significantly above the curves of the other imbalance-unaware methods (Fig. 5).

#### Precision, recall and F-score results at different score thresholds

To better understand the behavior of the different methods in the context of the prediction of non-coding common and rare disease associated variants, we performed a thorough analysis of the precision, recall, and F-score as a function of the score predicted by *hyperSMURF* and the other methods. In sum, with both Mendelian and GWAS data *hyperSMURF* shows the best F-score results in the full range of the prediction scores. This is mainly the consequence of the better *hyperSMURF* precision, while maintaining a recall comparable with that of the other methods (Supplementary Figs 2–5). Interestingly enough, the second best method is *GWAVA*, the only one among the compared methods that explicitly adopts imbalance-aware learning techniques. Detailed precision, recall and F-score results as a function of the score thresholds are presented in Supplementary Figs 2–5 and discussed in Supplementary Note 1.

#### Comparison of PR and ROC curves using precomputed scores

We performed also an experimental comparison between *hyperSMURF* and the other methods using pre-computed scores or scores obtained by pre-trained models available from the websites of *CADD*, *GWAVA*, *DeepSEA* and *Eigen*. The results in terms of AUROC and AUPRC with Mendelian data confirm previous results obtained by fully retraining the compared models: *hyperSMURF* achieves the best results in terms of AUPRC, followed by *GWAVA*, *Eigen*, *DeepSEA* and *CADD*, while the differences between the methods in terms of AUROC are less evident (Supplementary Fig. 6). As previously highlighted, we recall that in the context of highly imbalanced data sets AUPRC results are more informative and significant with respect to AUROC results^35, 36^.

#### *HyperSMURF* performance with respect to specific functional elements

Supplementary Table 8 shows the performance of *hyperSMURF* with Mendelian data considering variants located in different functional elements of non-coding regions (i.e. 5′UTR, 3′UTR, promoter, enhancer, etc). Results highlight that *hyperSMURF* achieves relatively good results for different categories of functional elements, although the performance was poorer for mutations in 3′UTRs and enhancers. Overall, the number of available annotations for each type of functional elements is not enough to allow reliable ML predictions for each specific class of functional elements.

### Performance of imbalance-aware and -unaware ML methods at different imbalance levels

In order to investigate the effect of imbalance in the functional prioritization of rare variants we performed testing with progressively imbalanced data. To this end we constructed, starting from the Mendelian data, four data sets with a fixed number of positives (all the available 406 positive examples) and a progressively increasing amount of negatives. The negative examples were sampled from the 14,755,199 negatives available in the Mendelian data. Sampling of negatives has been performed ensuring a balanced distribution across the cytoband-based CV partitions (constructed for the Mendelian data analysis). We first sorted all the variants according to their genomic position. Then we traversed the sorted set of variants and we extracted one negative for every *s* examples (where *s* is the step selection required to obtain the desired amount of negatives). By following this sampling strategy we ensure that while the genomic density of the sampled negatives varies across the cytobands their genomic coverage (in terms of genome regions spanning) is preserved. Given that the amount of variants is not equal across the available cytoband-based CV partitions we obtained four set of negatives containing 400, 4000, 39,930 and 394,957 variants with progressively increasing positive:negative imbalance ratios, approximately equal respectively to 1:1, 1:10, 1:100 and 1:1000. We finally performed a prediction test using an imbalance-unaware (*CADD*) versus an imbalance-aware (*hyperSMURF*) method. Performance were evaluated in terms of cytoband-aware 10-fold cross-validated AUPRC.

**Figure 6:**
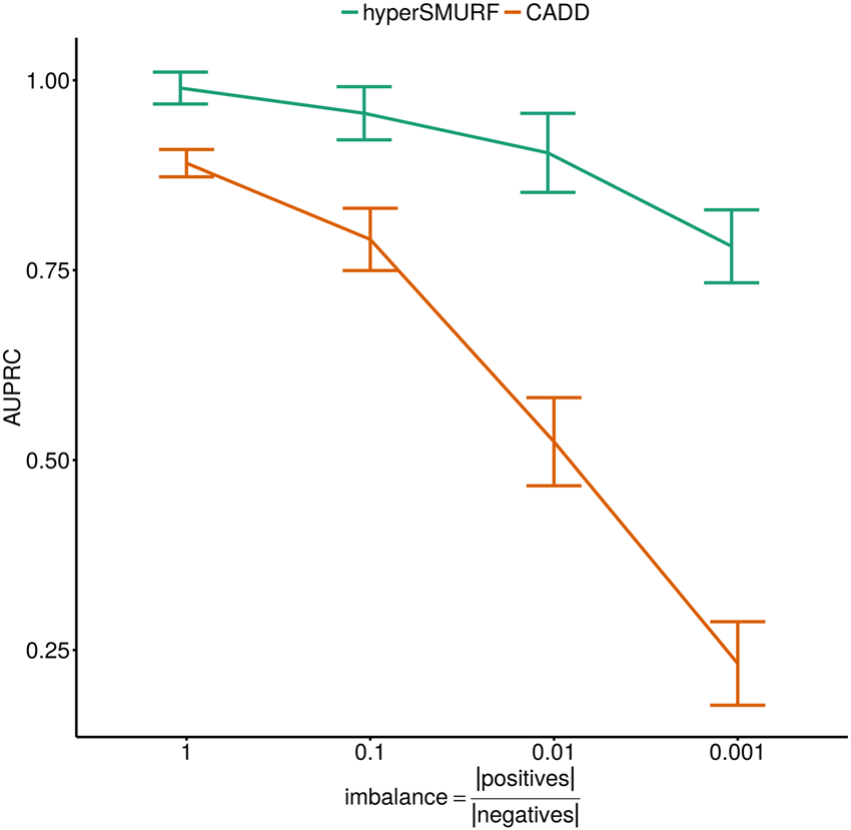
Performance at different imbalance levels. Area under the PR curve (AUPRC) results at different imbalance levels with imbalance-unaware (CADD) and imbalance-aware methods (*hyperSMURF*). The values on the X axis denote the ratio between positive and negative examples using Mendelian data. Error bars show, at each point, the cross-validated standard deviation.

The results shown in Fig. 6 further support the advantage of imbalance-aware ML methods in the prediction of rare genomic features. As expected, the imbalance unaware, supervised method *CADD* is very sensitive to the effect of the progressively increasing imbalance between positive and negative examples, while *hyperSMURF* only slightly decreases its performance when the imbalance grows. Moreover the difference between *CADD* and *hyperSMURF* is relatively small when the data are balanced and the curves progressively diverge at higher imbalance levels. This confirms that, when predicting rare disease-associated variants under real world settings where the size of positive examples is low for the underlying biology of the prediction problem, or the availability of high confidence positive examples is limited by experimental costs and time-consuming manual biocuration, imbalance-aware methods display a superior performance. As explained above and outlined in recent ML and computational biology literature^35, 36^, we note that the detection of the detrimental effect on prediction performance of highly imbalanced data is more evident when the AUPRC instead of the AUROC is used as performance evaluation metric (Supplementary Table 3).

### *HyperSMURF* can detect disease variants genomically close to non-disease variants

Pathogenic and non-deleterious variants that are genomically close to each other may share common genomic properties. From this standpoint the detection of a disease causing variant among its genomically close non-deleterious counterparts represents a computational challenge. To test the capability of *hyperSMURF* to distinguish a disease variant among its genomically close “negative” counterparts, we performed further experiments by selecting as negatives those variants being within 100, 500, or 1000 Kb distance from a positive, or being in the same topologically-associated domain (TAD). Moreover, for the evaluation of the performance we performed a “topologically-aware” cross-validation, by constructing a number of folds equal to the number of TADs or the number of 100, 500, or 1000 Kb “genomic windows” around each positive. Note that superposed windows have been merged in the same fold. In this way the performance evaluation metrics are calculated by ranking each positive over its “matched” negatives, and are not generally based on the overall ranking of all positive variants over all negative variants. This experimental setting requires months of computational time with standard stand-alone workstations. For this reason we run the task on a High Performance Cluster using 70 nodes at the same time, each one with 32 cores and 120 GB RAM. This massive parallelization reduced the computational time to two days.

Table 1 summarizes the results obtained with these negative selection strategies and the “topologically-aware” cross-validation. At first we note that the imbalance between positive and negatives, even if reduced with respect to the previous experimental setting, remains an important issue. Indeed with Mendelian data we have imbalance ratios ranging from about 1:300 to 1:2800, and with GWAS data from 1:80 to 1:400. The overall results confirm that *hyperSMURF* is able to correctly detect positives among its “neighborhood” negatives. With GWAS data the results are slighlty worse than those obtained with the previous experimental setting (see paragraph “Area Under the Precision-Recall Curve (AUPRC) results”), but the AUPRC is relatively high and the AUROC is close to 1. With Mendelian data the performance, independently of the negative strategy selection, are also better than in the previous experimental set-up, with a significant increment of the AUPRC, likely due to the significant reduction of the imbalance ratio (Fig. 4 and Table 1).

**Table 1:** Performance of *hyperSMURF* with different selection strategies for negatives.

Neg. selection	imb.ratio	n.folds	AUPRC	AUROC
**Mendelian data**				
±100 Kb	1:302	116	0.7071	0.9805
±500 Kb	1:1432	116	0.6279	0.9802
±1000 Kb	1:2765	111	0.6161	0.9786
TAD	1:1406	125	0.6123	0.9803
**GWAS data**				
±100 Kb	1:80	1402	0.6488	0.9840
±500 Kb	1:277	723	0.4796	0.9841
±1000 Kb	1:409	413	0.4213	0.9851
TAD	1:269	1196	0.4792	0.9838

The first column represents the size of the “genomic window” used to select negatives around each positive or the “TAD-based” negative selection strategy; the second column reports the imbalance between positive and negative examples; the third the number of folds of the “topologically-aware” cross-validation, while the last two columns show the estimated AUPRC and AUROC.

## Discussion

The experimental results show that imbalance-aware methods significantly outperform imbalance-unaware ML methods for the prediction of regulatory non-coding Mendelian and common disease associated variants. *HyperSMURF*, a method specifically designed to deal with imbalanced data, achieves the best results with both Mendelian and GWAS data, i.e. in the context of different problems involving the prediction of rare genetic variants, and using also different sets of input features: conservation, transcription and regulation features as well as G/C content and population-based-features for Mendelian data, and genomic features directly extracted from the DNA sequence through deep convolutional networks for GWAS regulatory hits. Despite these differences, both prediction problems face the challenge of a substantial imbalance between “positive” and “negative” examples.

Moreover the experiments with Mendelian data artificially balanced and then progressively imbalanced by increasing the number of “negative” variants, further show that in a real scenario the imbalance-awareness of ML methods are essential for the detection of rare categories of genetic variation in the human genome. Especially in Mendelian disease genomes we expect only one or a few causative mutations directly linked to the disease. But a whole genome contains millions of variants that can only be prefiltered to a few thousands using allele frequencies in populations^10^. Therefore the imbalance actually occurs in real data and imbalance-aware methods are specifically designed to deal with this problem.

It is also striking that the second best method was determined to be *GWAVA*, the only one among published state-of-the-art methods that employs undersampling imbalance-aware learning strategies. In contrast to *GWAVA*, *hyperSMURF* additionally introduces oversampling techniques to add new synthetic positive examples very similar to the original ones, thus enlarging the set of deleterious variants available for training the base learners. Moreover partitioning of the data allows a large coverage of the available training data, while maintaining relatively small training data sets, and finally the hyper-ensemble approach allows to achieve robust and accurate base learners. The impact of each of these components on the learning behavior of *hyperSMURF* are experimentally analyzed in the Supplementary Note 2 and Supplementary Figs 7–9, showing that the synergy between partitioning, undersampling, oversampling, ensembling and hyper-ensembling techniques is a key factor for improving the performance of *hyperSMURF* in predicting deleterious non-coding variants.

In addition experimental results show that *hyperSMURF* is able to distinguish disease variants genomically close to non-disease variants: AUPRC results achieved with this experimental setting are sometimes also better than those obtained with the experimental set-up that includes all the negatives and the overall ranking of all positives vs all negatives. This is not so surprising, since despite the genomic similarities between a positive and its “matched” negatives, “TAD-aware” and “genomic-window-aware” experimental settings lead to less imbalanced prediction tasks.

A limitation of the proposed approach is represented by the large number of learning parameters. Indeed the proper choice of their values is not always straightforward and may have a certain impact on the accuracy of the algorithm (see Supplementary Note 2 for more details). More precisely, *hyperSMURF* is characterized by different learning parameters of both the hyper-ensemble (the number of partitions *n*, the oversampling and undersampling factors *f* and *m*–see Methods section) and of the random forests (e.g. the number of trees of the ensemble or the number of features to be randomly selected at each node of the decision trees). We provide a set of default parameters that worked reasonably in our experiments in Supplementary Table 4. Moreover we analyzed the impact of the different learning parameters on the learning behavior of *hyperSMURF* and we propose also an experimental procedure to automatically tune the parameters by learning them from the data (see Supplementary Section 2 and Supplementary Table 5 for more details).

In sum, our method is characterized by the following properties, well-suited for the prediction of deleterious variants in imbalanced experimental settings: a) Under- and oversampling techniques allow the construction of balanced training sets, thus avoiding the bias toward the non-deleterious genetic variants, and lead also to diverse base learners, a well-known factor for the success of ensemble methods^21^; b) The relatively small size of the training data is counterbalanced by the partitioning of the data and by the ensemble approach, by which our method can learn from a large set of the available data, thus improving the coverage of the available training data; c) Predictions are provided by a committee of RFs (i.e. an ensemble of ensembles of random trees–hyper-ensemble approach), thus improving the robustness and the accuracy of the predictions; d) the relatively small size of the training examples scales nicely with large amounts of data (with genetic variants we usually have millions of examples) and the ensemble approach allows a relatively easy parallel implementation of the algorithm, as shown by our *hyperSMURF* package freely downloadable from the CRAN public repository.

We think that this method and associated software tools could be helpful for biologists and physicians to discover trait and disease-associated variants in contexts characterized by imbalanced genomic data. Moreover our work shows that imbalance-aware ML is a central issue for the development of methods for the prediction of deleterious variants in non-coding regions.

## Electronic supplementary material

Supplementary Information

## References

[1] Ward LD, Kellis M (2012). Interpreting noncoding genetic variation in complex traits and human disease. Nat. Biotechnol..

[2] Veltman JA, Lupski JR (2015). From genes to genomes in the clinic. Genome Med..

[3] Ritchie, G. & Flicek, P. Functional Annotation of Rare Genetic Variants in *Assessing Rare Variation in Complex Traits* (eds Zeggini, E. & Morris, A.) 57–70 (Springer New York, 2015). 30964620

[4] Kircher M (2014). A general framework for estimating the relative pathogenicity of human genetic variants. Nat. Genet..

[5] Ritchie GRS, Dunham I, Zeggini E, Flicek P (2014). Functional annotation of noncoding sequence variants. Nat. Methods.

[6] Zhou J, Troyanskaya OG (2015). Predicting effects of noncoding variants with deep learning-based sequence model. Nat. Methods.

[7] Shihab HA (2015). An integrative approach to predicting the functional effects of non-coding and coding sequence variation. Bioinformatics.

[8] Lee D (2015). A method to predict the impact of regulatory variants from dna sequence. Nat. Genet..

[9] Ionita-Laza I, McCallum K, Xu B, Buxbaum JD (2016). A spectral approach integrating functional genomic annotations for coding and noncoding variants. Nat. Genet..

[10] Smedley D (2016). A Whole-Genome Analysis Framework for Effective Identification of Pathogenic Regulatory Variants in Mendelian Disease. Am. J. Hum. Genet..

[11] Forbes SA (2015). Cosmic: exploring the world’s knowledge of somatic mutations in human cance. r. Nucleic Acids Res..

[12] Ma M (2015). Disease-associated variants in different categories of disease located in distinct regulatory elements. BMC Genomics.

[13] Visscher PM (2012). Five years of GWAS discovery. Am. J. Hum. Genet..

[14] Edwards SL (2013). Beyond GWASs: Illuminating the Dark Road from Association to Function.. Am. J. Hum. Genet..

[15] Gordon T, Lyonnet S (2014). Enhancer mutations and phenotype modularity. Nat. Genet..

[16] Cortes C, Vapnik V (1995). Support vector networks. Mach. Learn..

[17] Bishop, C. M. *Neural Networks for Pattern Recognition* (Oxford University Press, 1995).

[18] Galar M, Fernandez A, Barrenechea E, Bustince H, Herrera F (2012). A review on ensembles for the class imbalance problem: Bagging-, boosting-, and hybrid-based approaches. Syst. Man, Cybern. Part C Appl. Rev. IEEE Trans..

[19] Welter D (2014). The NHGRI GWAS Catalog, a curated resource of SNP-trait associations. Nucleic Acids Res..

[20] Chawla, N. V., Bowyer, K. W., Hall, L. O. & Kegelmeyer, W. P. SMOTE: Synthetic Minority Over-sampling Technique. *J*. *Artif*. *Intell*. *Res*. 321–357 (2002).

[21] Kuncheva, L. *Diversity in Classifier Ensembles*, 247–289 (John Wiley & Sons, Inc., 2014).

[22] Kuncheva, L. *Combining Pattern Classifiers*: *Methods and Algorithms*, *2nd edition* (Wiley-Interscience, New York, 2014).

[23] Dietterich, T. Ensemble methods in machine learning in *Multiple Classifier Systems* (eds Kittler, J. & Roli, F.) 1–15 (Springer-Verlag, 2000).

[24] Re, M. & Valentini, G. Ensemble methods: a review in *Advances in Machine Learning and Data Mining for Astronomy* (ed. Kumar, V.) 563–594 (Chapman & Hall, 2012).

[25] Breiman L (2001). Random forests. Mach. Learn..

[26] Goldstein B, Polley E, Briggs F (2011). Random forests for genetic association studies. Stat. Appl. Genet. Mol. Biol..

[27] Liu X, Wu J, Zhou Z (2009). Exploratory undersampling for class-imbalance learning. IEEE Trans. Syst. Man, Cybern. Part B Cybern..

[28] He H, Garcia E (2009). Learning from imbalanced data. IEEE Trans. Knowl. Data Eng..

[29] Paten B (2008). Genome-wide nucleotide-level mammalian ancestor reconstruction. Genome Res..

[30] Paten B, Herrero J, Beal K, Fitzgerald S, Birney E (2008). Enredo and pecan: genome-wide mammalian consistency-based multiple alignment with paralogs. Genome Res..

[31] Abecasis GR (2012). An integrated map of genetic variation from 1,092 human genomes. Nature.

[32] Fan R, Chang K, Hsieh C, Wang X, Lin C (2008). LIBLINEAR: A library for large linear classification. J. Mach. Learn. Res..

[33] Hall M (2009). The WEKA data mining software. ACM SIGKDD Explor. Newsl..

[34] DeLong E, DeLong D, Clarke-Pearson D (1988). Comparing the areas under two or more correlated receiver operating characteristic curves: A nonparametric approach. Biometrics.

[35] Saito T, Rehmsmeier M (2015). The precision-recall plot is more informative than the roc plot when evaluating binary classifiers on imbalanced datasets. PLoS One.

[36] Davis, J. & Goadrich, M. The relationship between precision-recall and roc curves in *Proceedings of the 23rd International Conference on Machine Learning* 233–240 (ACM, 2006).

[37] Pollard KS, Hubisz MJ, Rosenboom K, Siepel A (2010). Detection of non-neutral substitution rates on Mammalian phylogenies. Genome Res..

