# Supplementary material for: Imbalance-Aware Machine Learning for Predicting Rare and Common Disease-Associated Non-Coding Variants
Source: Sci Rep. 2017 Jun 7;7:2959. doi: 10.1038/s41598-017-03011-5 (PMC5462751; doi:10.1038/s41598-017-03011-5)
Supplement: Supplementary file 1 — Supplementary Information [file 41598_2017_3011_MOESM1_ESM.pdf]

Imbalance-Aware Machine Learning for Predicting Rare and Common Disease-Associated Non-Coding Variants - Supplementary Information

Max Schubach[1], **Matteo Re**[2], **Peter N Robinson**[1,3,4], **and Giorgio Valentini**[2,*]

[1]Institute for Medical and Human Genetics, Charité – Universitätsmedizin Berlin, Augustenburger Platz 1, 13353 Berlin, Germany
[2]Anacletolab, Dipartimento di Informatica, Università degli Studi di Milano, Via Comelico 39, 20135 Milan, Italy
[3]The Jackson Laboratory for Genomic Medicine, 10 Discovery Drive. Farmington, CT 06032, USA
[4]Institute for Systems Genomics, University of Connecticut, Farmington, CT 06032, USA
[*]valentini@di-unimi.it

Contents

Page

Supplementary Tables . . . 2
Supplementary Table 1. Negative training data of the Mendelian dataset. . . . 2
Supplementary Table 2. Genomic attributes for the regulatory Mendelian dataset. . . . 3
Supplementary Table 3. Comparison of imbalance-unaware and imbalance-aware methods with progressively imbalanced data. . . . 4
Supplementary Table 4. *HyperSMURF* default parameters. . . . 4
Supplementary Table 5. Impact of *hyperSMURF* components on its overall performance. . . . 4
Supplementary Table 6. Most informative Mendelian genomic features according to a univariate regression model. . . 5
Supplementary Table 7. Informative GWAS genomic features according to a univariate regression model. . . . 6
Supplementary Table 8. *HyperSMURF* performance with respect to specific functional elements . . . 6
Supplementary Figures . . . 7
Supplementary Figure 1. Comparison across methods of ROC curves. . . . 7
Supplementary Figure 2. Precision, recall, and F-score comparison across methods with GWAS data. . . . 8
Supplementary Figure 3. GWAS data: precision, recall, and F-measure in the highest range of the normalized score. . 9
Supplementary Figure 4. Performance comparison across methods with Mendelian data. . . . 10
Supplementary Figure 5. Mendelian data: precision, recall, and F-measure in the highest range of the normalized score. 11
Supplementary Figure 6. Comparison of ROC and PR curves using precomputed scores. . . . 12
Supplementary Figure 7. *HyperSMURF* parameter tuning with fixed undersampling factor. . . . 13
Supplementary Figure 8. *HyperSMURF* parameter tuning with fixed oversampling factor. . . . 14
Supplementary Figure 9. Tuning of Random Forest parameters. . . . 15
Supplementary Figure 10. Characteristics of the two sets of DeepSEA features. . . . 15
Supplementary Notes. . . . 16
Supplementary Note 1: Supplementary Experimental results . . . 16
Supplementary Note 2: Experimental analysis and automatic tuning of the parameters of *HyperSMURF*. . . . 17
Supplementary Note 3: The *hyperSMURF* software R package. . . . 18
References **21**

Supplementary Tables

Supplementary Table 1. Negative training data of the Mendelian dataset. Distribution of variant categories for single nucleotide positions in *Homo sapiens* that differ from the inferred sequence of the last common primate ancestor. An asterisk (*) marks variant categories that were used as negatives. Variants were chosen from the Sequence Ontology[1] categories NON_CODING_TRANSCRIPT_INTRON_VARIANT, CODING_TRANSCRIPT_INTRON_VARIANT, FIVE_PRIME_UTR_VARIANT, THREE_PRIME_UTR_VARIANT, UPSTREAM_GENE_VARIANT, DOWNSTREAM_GENE_VARIANT, INTERGENIC_VARIANT, TF_BINDING_SITE_VARIANT, REGULATORY_REGION_VARIANT, CONSERVED_INTRON_VARIANT, INTRAGENIC_VARIANT, CONSERVED_INTERGENIC_VARIANT, INTRON_VARIANT. Variants were defined at positions in which the human genome differs from the inferred genome sequence of the last common primate ancestor and annotated using Jannovar[2] version 0.14 using transcript definitions from the NCBI Reference Sequence Database[3] (annotation release 105).

Category	All	High quality	Fixed	High-quality & Fixed
CDS	49,599	44,885	43,420	38,706
CDS (syn)	57,708	52,656	52,189	47,137
Unclassified sequence variant	11,408	10,675	11,408	10,675
Splice	12,520	12,553	12,430	11,218
5’ UTR	764,719	711,934	692,943	640,158*
3’ UTR	121,014	112,740	109,034	100,760*
Intron	5,954,014	5,600,983	5,383,124	5,030,093*
Upstream/Downstream	224,128	198,554	203,737	178,163*
Noncoding (exon)	67,704	58,236	62,038	52,570
Noncoding (intron)	858,848	782,486	782,720	706,358*
Intergenic	9,908,106	8,989,024	9,018,749	8,099,667*
Total	18,029,768	16,574,726	16,371,792	14,915,505

Supplementary Table 2. Genomic attributes for the regulatory Mendelian dataset. Genomic attributes used for training the regulatory Mendelian dataset. The source of the data is shown as a UCSC Genome Browser Table or as a URL.

Attribute	Description
GCContent	GC-content in a window of ± 75 nt
CpGperGC	Percentage of CpG island that is C or G.<br>UCSC table `cpgIslandExt`
CpGperCpG	Percentage of CpG island that is CpG.<br>UCSC table `cpgIslandExt`
CpGobsExp	Ratio of observed to expected CpG in CpG island.<br>UCSC table `cpgIslandExt`
priPhyloP46way	Primate PhyloP score.<br>`http://hgdownload.soe.ucsc.edu/goldenPath/hg19/phyloP46way/primates`
verPhyloP46way	Vertebrate PhyloP.<br>`http://hgdownload.soe.ucsc.edu/goldenPath/hg19/phyloP46way/vertebrate`
mamPhyloP46way	Mammalian PhyloP score.<br>`http://hgdownload.soe.ucsc.edu/goldenPath/hg19/phyloP46way/placentalMammals`
priPhastCons46way	Primate PhastCons conservation score<br>`http://hgdownload.soe.ucsc.edu/goldenPath/hg19/phastCons46way/primates`
verPhastCons46way	Vertebrate PhastCons conservation score<br>`http://hgdownload.soe.ucsc.edu/goldenPath/hg19/phastCons46way/vertebrate`
mamPhastCons46way	Mammalian PhastCons conservation score<br>`http://hgdownload.soe.ucsc.edu/goldenPath/hg19/phastCons46way/placentalMammals`
GerpRS	GERP++ element score<br>`http://mendel.stanford.edu/SidowLab/downloads/gerp/hg19.GERP_elements.tar.gz`
GerpRSpv	GERP++ element p-Value<br>`http://mendel.stanford.edu/SidowLab/downloads/gerp/hg19.GERP_elements.tar.gz`
EncH3K27Ac	Maximum ENCODE H3K27 acetylation level<br>`http://hgdownload.cse.ucsc.edu/goldenPath/hg19/encodeDCC/wgEncodeRegMarkH3k27ac`
EncH3K4Me1	Maximum ENCODE H3K4 methylation level<br>`http://hgdownload.cse.ucsc.edu/goldenPath/hg19/encodeDCC/wgEncodeRegMarkH3k4me1`
EncH3K4Me3	Maximum ENCODE H3K4 trimethylation level<br>`http://hgdownload.cse.ucsc.edu/goldenPath/hg19/encodeDCC/wgEncodeRegMarkH3k4me3`
DnaseClusteredHyp	DnaseClustered V3 hypersensitivity score<br>`http://hgdownload.cse.ucsc.edu/goldenPath/hg19/encodeDCC/wgEncodeRegDnaseClustered`
DnaseClusteredScore	Number of DnaseClustered V3 hypersensitive cells<br>`http://hgdownload.cse.ucsc.edu/goldenPath/hg19/encodeDCC/wgEncodeRegDnaseClustered`
fantom5Perm	FANTOM 5 permissive enhancers<br>`http://enhancer.binf.ku.dk/presets/permissive_enhancers.bed`
fantom5Robust	FANTOM5 robust enhancers<br>`http://enhancer.binf.ku.dk/presets/robust_enhancers.bed`
numTFBSConserved	Number of overlapping transcription factor binding sites.<br>UCSC table `tfbsConsSites`
rareVar	Number of rare 1000 Genome variants ($\leq 0.5\%$ AF) in a window of ± 500 nt
commonVar	Number of common 1000 Genome variants ($> 0.5\%$ AF) in a window of ± 500 nt
fracRareCommon	Ratio of rare to common variants
ISCApath	Overlapping ISCA CNVs<br>`http://www.ncbi.nlm.nih.gov/dbvar/studies/nstd75`<br>`http://www.ncbi.nlm.nih.gov/dbvar/studies/nstd46`<br>`http://www.ncbi.nlm.nih.gov/dbvar/studies/nstd37`
dbVARCount	Overlapping dbVAR CNVs<br>`ftp://ftp.ncbi.nlm.nih.gov/pub/dbVar/data/Homo_sapiens/`<br>`by_assembly/GRCh37.p13/gvf/GRCh37.p13.remap.all.germline.ucsc.gvf.gz`
DGVCount	Overlapping DGV CNVs<br>`http://dgv.tcag.ca/dgv/app/downloads?ref=GRCh37/hg19`

Supplementary Table 3. Comparison of imbalance-unaware and imbalance-aware methods with progressively imbalanced data. Imbalance-unaware (CADD) and imbalance-aware (*hyperSMURF*) area under the precision/recall curve (AUPRC) and area under the ROC curve (AUROC) results using cytoband-aware 10-fold cross-validation with Mendelian data. Imbalance ratio represents the ratio between positive examples (Mendelian mutations) and negatives (background non-deleterious variants).

Imbalance ratio	AUPRC		AUROC	
	CADD	*HyperSMURF*	CADD	*HyperSMURF*
1	0.8908	0.9897	0.8702	0.9908
0.1	0.7905	0.9565	0.8946	0.9891
0.01	0.5242	0.9044	0.8896	0.9904
0.001	0.2323	0.7814	0.8534	0.9912

Supplementary Table 4. *HyperSMURF* default parameters. Default values and sets of parameter values explored for the automatic tuning of *hyperSMURF*. The item "Random tree features" refers to the number of randomly selected features at each step of the construction of the inductive trees that constitute the base learners of the random forests. As a general default on larger datasets we propose $d = \left\lfloor \sqrt{|features|} \right\rfloor$. More details about the parameters of *hyperSMURF* are available in the Supplementary Note 1.

Parameter	Description	Default	Parameter values used for optimization
n	Number of partitions	100	$10, 25, 50, 75, 100, 150, 200$
f	SMOTE oversampling factor	2	$0.5, 1, 1.5, 2, 2.5, 3$
k	SMOTE k-nearest neighbor	5	5
m	Undersampling factor	3	$1, 2, 3$
t	Forest size	10	$5, 10, 20, 30, 50, 75, 100$
d	Random tree features	5	$3, 4, 5, 6, 7, 10$

Supplementary Table 5. Impact of *hyperSMURF* components on its overall performance. Area under the ROC curve (AUROC), area under the precision/recall curve (AUPRC) and AUROC of the top 100, 500 and 1000 variants ($AUROC_{100}$, $AUROC_{500}$ and $AUROC_{1000}$) with the Mendelian data. *HyperSMURF* std is the full *HyperSMURF* algorithm; *HyperSMURF* no-over is *HyperSMURF* with no oversampling; *HyperSMURF* no-par is *HyperSMURF* with no partitions, i.e. without the hyper-ensemble approach (no partitioning of the training data is performed); RF is the classical Random Forest ensemble. In every setting a subsampling of the majority (negative) class to three times the cardinality of the minority class (positives) has been performed. Other parameters were set to default (see Supplementary Table 4).

Algorithm	AUPRC	AUROC	$AUROC_{100}$	$AUROC_{500}$	$AUROC_{1000}$
HyperSMURF std	0.4266	0.9900	0.6962	0.8034	0.8519
HyperSMURF no-over	0.3925	0.9915	0.6457	0.7969	0.8475
HyperSMURF no-par	0.0287	0.9870	0.0000	0.5000	0.5000
RF	0.0149	0.9891	0.0000	0.5000	0.5000

Supplementary Table 6. Most informative Mendelian genomic features according to a univariate regression model. Results are computed by 10-fold cytoband-aware cross-validation using a logistic regression model with Mendelian data. Experiment is repeated 100 times and AUPRC and AUROC means with the standard deviations are listed. Genomic features are ranked according to the estimated AUPRC. The source of the data for the considered genomic features are listed in Supplementary Table 2.

Genomic feature	AUPRC	AUROC
mamPhyloP46way	0.24018 ± 0.01600	0.92077 ± 0.00025
verPhyloP46way	0.11705 ± 0.00635	0.92450 ± 0.00028
priPhyloP46way	0.02750 ± 0.00541	0.96333 ± 0.00036
priPhastCons46way	0.00951 ± 0.00965	0.89562 ± 0.00316
mamPhastCons46way	0.00157 ± 0.00020	0.85717 ± 0.00171
verPhastCons46way	0.00121 ± 0.00019	0.84922 ± 0.00177
DnaseClusteredHyp	0.00101 ± 0.00032	0.73808 ± 0.00414
CpGperCpG	0.00096 ± 0.00040	0.61934 ± 0.00257
CpGobsExp	0.00093 ± 0.00036	0.61912 ± 0.00286
CpGperGC	0.00081 ± 0.00019	0.61898 ± 0.00235
numTFBSConserved	0.00062 ± 0.00011	0.62967 ± 0.01099
EncH3K4Me3	0.00058 ± 0.00004	0.81133 ± 0.01060
GerpRS	0.00039 ± 0.00004	0.84027 ± 0.00724
DnaseClusteredScore	0.00032 ± 0.00005	0.73790 ± 0.00222
GCContent	0.00032 ± 0.00002	0.82201 ± 0.00091
rareVar	0.00024 ± 0.00010	0.50697 ± 0.00233
EncH3K27Ac	0.00024 ± 0.00001	0.79388 ± 0.00877
EncH3K4Me1	0.00006 ± 0.00000	0.73490 ± 0.00819
fracRareCommon	0.00005 ± 0.00000	0.67170 ± 0.00201
commonVar	0.00004 ± 0.00000	0.58627 ± 0.00563
ISCApath	0.00004 ± 0.00000	0.50986 ± 0.01101
GerpRSpv	0.00004 ± 0.00000	0.49735 ± 0.02040
fantom5Perm	0.00003 ± 0.00000	0.49468 ± 0.02366
fantom5Robust	0.00003 ± 0.00000	0.49362 ± 0.02293
dbVARCount	0.00002 ± 0.00000	0.44047 ± 0.03022
DGVCount	0.00002 ± 0.00000	0.44047 ± 0.03022

Supplementary Table 7. Informative GWAS genomic features according to a univariate regression model. Results are computed by 10-fold cytoband-aware cross-validation using a logistic regression model with GWAS data. Experiment is repeated 100 times and AUPRC and AUROC means with the standard deviations are listed. Genomic features are ranked according to the estimated AUPRC and the ten best and ten less informative features are listed.

Genomic feature	AUPRC	AUROC
PhyloP	0.36978 ± 0.01004	0.76382 ± 0.00019
PhastCons	0.04262 ± 0.00332	0.93689 ± 0.00094
HepG2|Sin3Ak-20|None|PDIFF	0.00415 ± 0.00006	0.51082 ± 0.00137
HeLa-S3|TBP|None|PDIFF	0.00414 ± 0.00010	0.50653 ± 0.00381
PFSK-1|FOXP2|None|PDIFF	0.00392 ± 0.00006	0.51070 ± 0.00329
HeLa-S3|GTF2F1|None|PDIFF	0.00391 ± 0.00009	0.50962 ± 0.00636
HepG2|Mxi1|None|PDIFF	0.00382 ± 0.00019	0.50738 ± 0.00276
A549|ETS1|EtOH_0.02pct|PDIFF	0.00381 ± 0.00012	0.51528 ± 0.00577
HepG2|MYBL2|None|PDIFF	0.00373 ± 0.00010	0.51412 ± 0.00084
GM12878|MAZ|None|PDIFF	0.00373 ± 0.00009	0.51408 ± 0.00264
⋮	⋮	⋮
NHDF-Ad|H3K9me3|None|PDIFF	0.00137 ± 0.00004	0.52076 ± 0.01015
NT2-D1|ZNF274|None|PDIFF	0.00137 ± 0.00003	0.50806 ± 0.00856
H1-hESC|H3K9me3|None|PDIFF	0.00136 ± 0.00003	0.51356 ± 0.00811
GM12878|ZNF274|None|PDIFF	0.00134 ± 0.00002	0.50384 ± 0.00432
HeLa-S3|BRF2|None|PDIFF	0.00132 ± 0.00004	0.49894 ± 0.00515
GM08714|ZNF274|None|PDIFF	0.00131 ± 0.00001	0.49587 ± 0.00388
HeLa-S3|SPT20|None|PDIFF	0.00130 ± 0.00003	0.50425 ± 0.00888
K562|KAP1|None|PDIFF	0.00129 ± 0.00001	0.50126 ± 0.00326
U2OS|SETDB1|None|PDIFF	0.00129 ± 0.00001	0.49855 ± 0.00371
K562|SETDB1|MNaseD|PDIFF	0.00127 ± 0.00001	0.48961 ± 0.00455

Supplementary Table 8. *HyperSMURF* performance with respect to specific functional elements. *HyperSMURF* performances with Mendelian data considering variants located in different functional elements of non-coding regions. The number of variants in the specific element is shown under #variants. $AUROC_{100}$ is the AUROC computed considering only the top ranked 100 variants; $AUROC_{500}$ and $AUROC_{1000}$ are computed in a similar way.

Functional element	#variants	AUPRC	AUROC	$AUROC_{100}$	$AUROC_{500}$	$AUROC_{1000}$
5’UTR	133	0.329	0.997	0.665	0.811	0.885
3’UTR	38	0.009	0.943	0.614	0.765	0.657
Enhancer	37	0.074	0.999	0.434	0.673	0.837
Promoter	130	0.185	0.991	0.589	0.785	0.815
microRNA	5	0.228	1.000	0.359	0.871	0.936
RNA component	60	0.407	1.000	0.832	0.833	0.874

Supplementary Figures

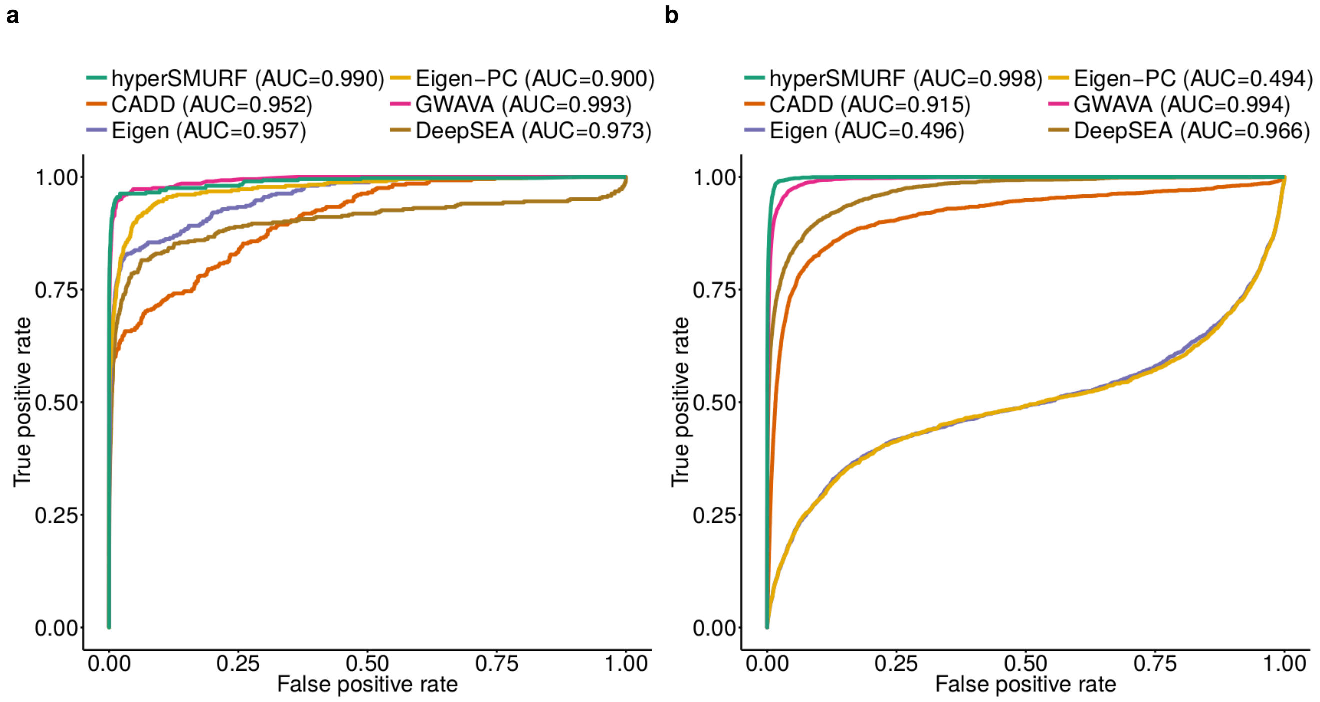

Supplementary Figure 1. Comparison across methods of ROC curves. a: ROC curves of the methods with Mendelian data; **b:** ROC curves with the regulatory GWAS hits.

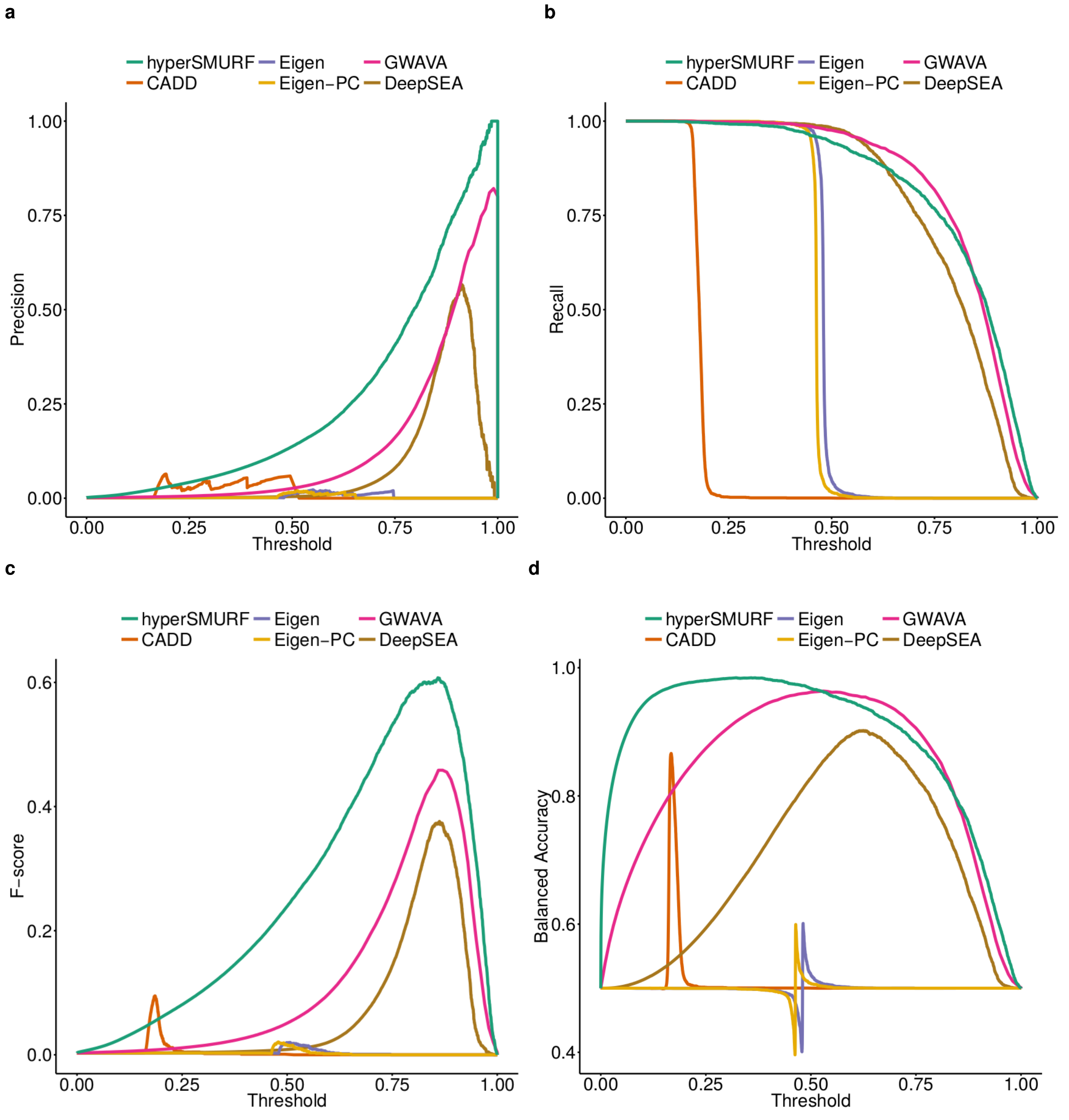

Supplementary Figure 2. Precision, recall, and F-score comparison across methods with GWAS data. *HyperSMURF*, CADD, Eigen, Eigen-PC, GWAVA, and DeepSEA performance, by varying the normalized score threshold. **a:** precision, **b:** recall, **c:** F-score, and **d:** balanced accuracy.

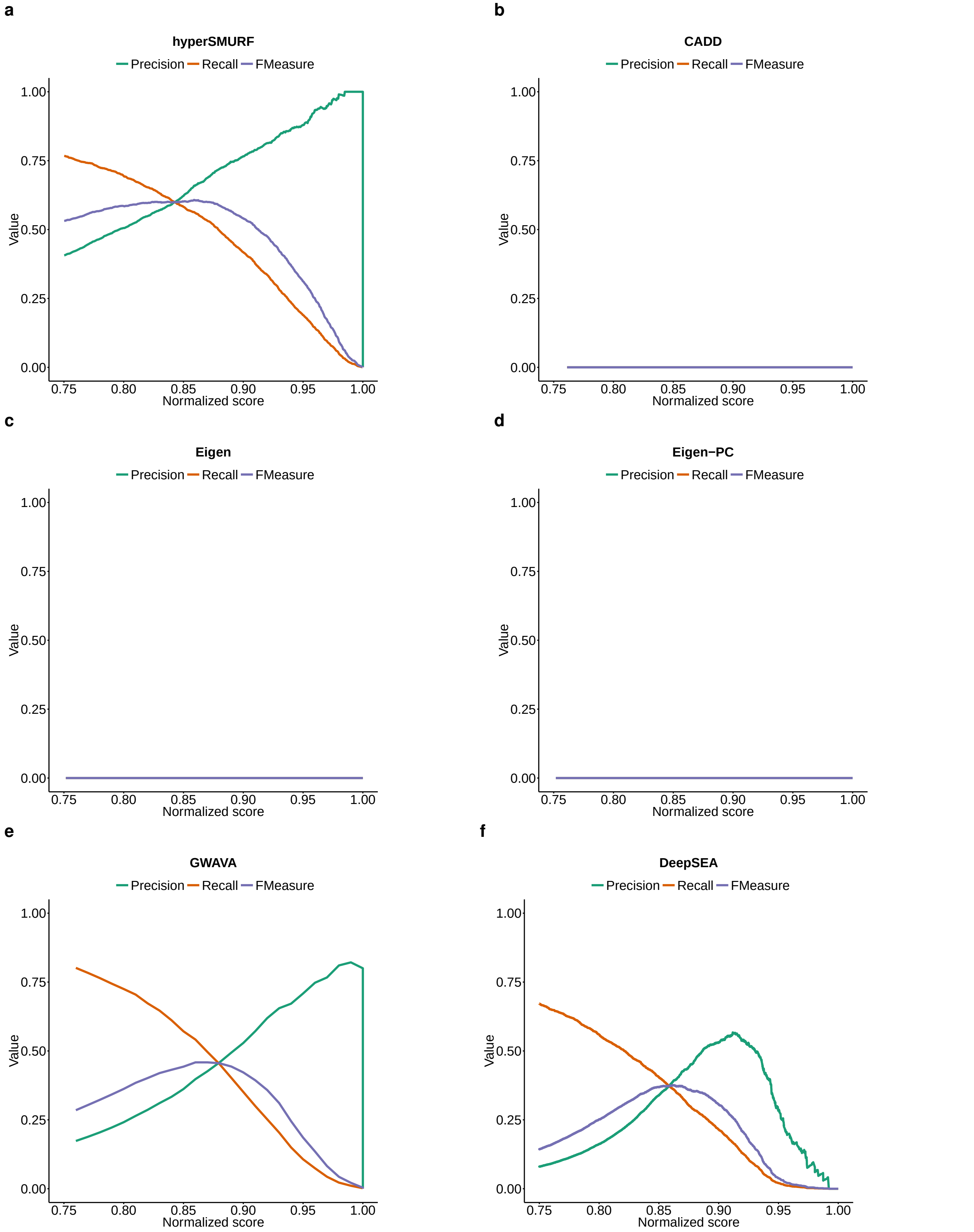

Supplementary Figure 3. GWAS data: precision, recall, and F-measure in the highest range of the normalized score. Details of precision, recall and F-score as a function of $[0.75, 1]$ values of the normalized scores. **a:** *HyperSMURF* **b:** CADD **c:** Eigen **d:** Eigen-PC **e:** GWAVA **f:** DeepSEA.

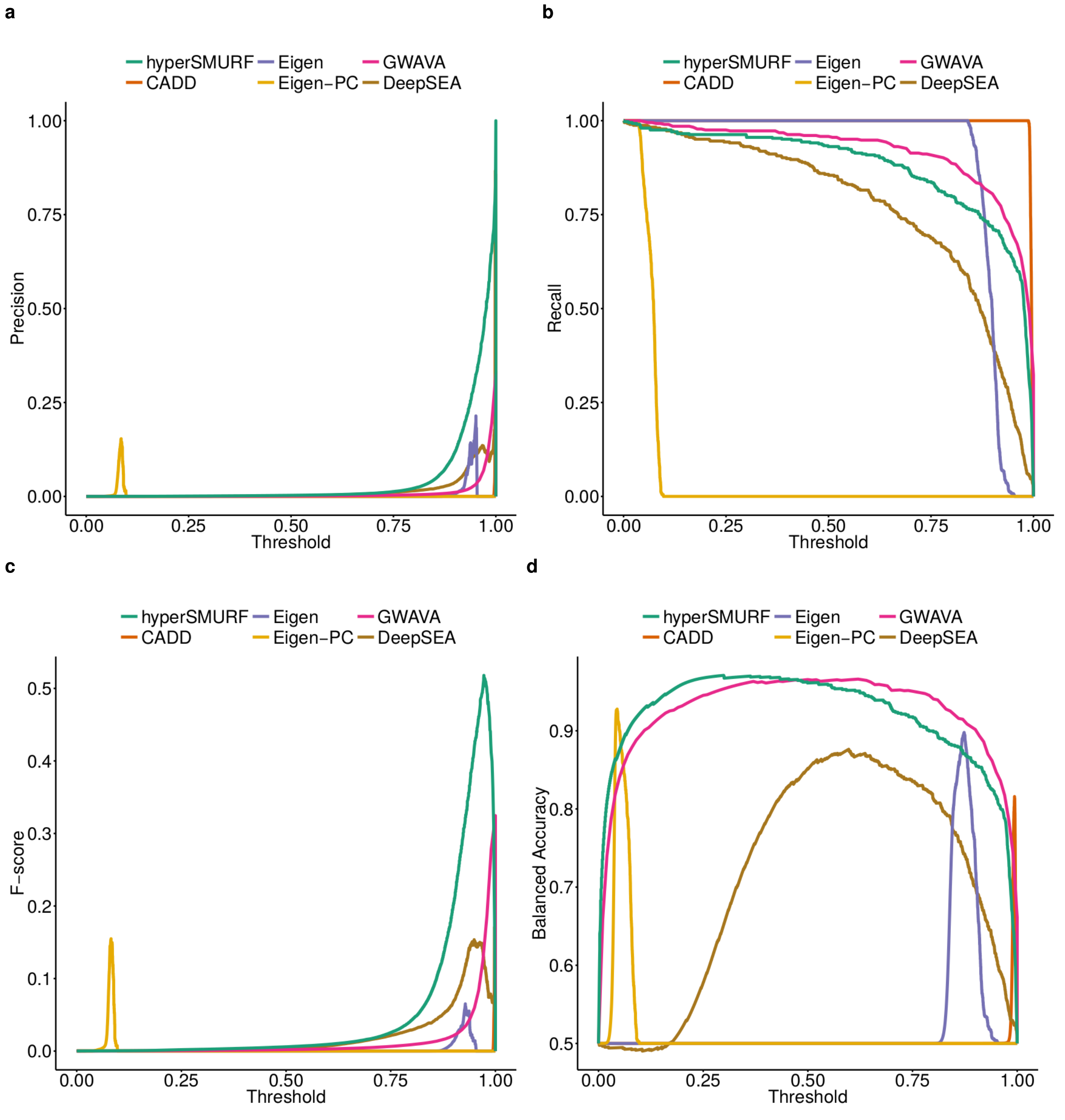

Supplementary Figure 4. Performance comparison across methods with Mendelian data. *HyperSMURF*, CADD, Eigen, Eigen-PC, GWAVA, and DeepSEA results by varying the normalized score threshold. **a:** precision, **b:** recall, **c:** F-score, and **d:** balanced accuracy.

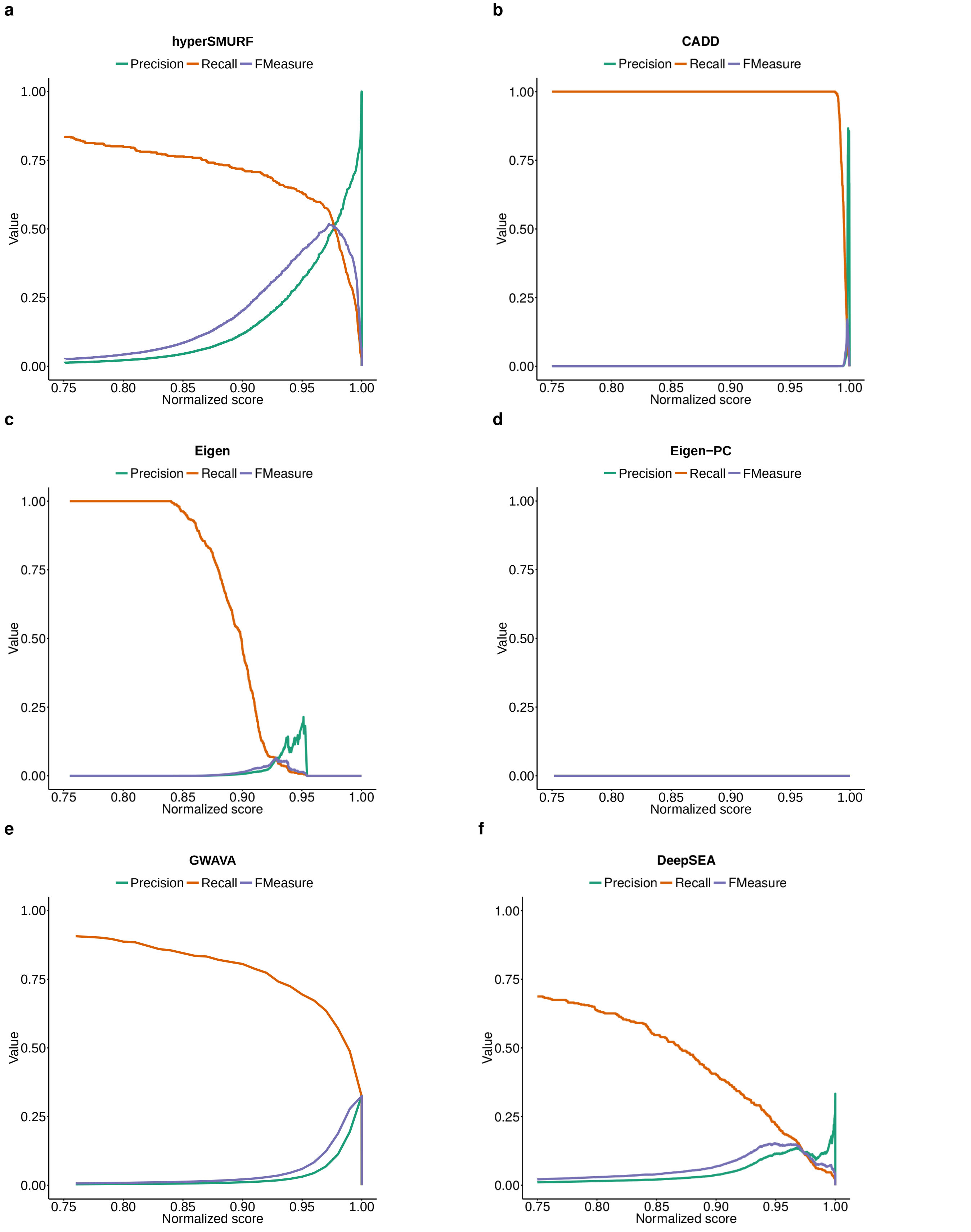

Supplementary Figure 5. Mendelian data: precision, recall, and F-measure in the highest range of the normalized score. Details of precision, recall and F-score as a function of $[0.75, 1]$ values of the normalized scores. **a:** *HyperSMURF* **b:** CADD **c:** Eigen **d:** Eigen-PC **e:** GWAVA **f:** DeepSEA.

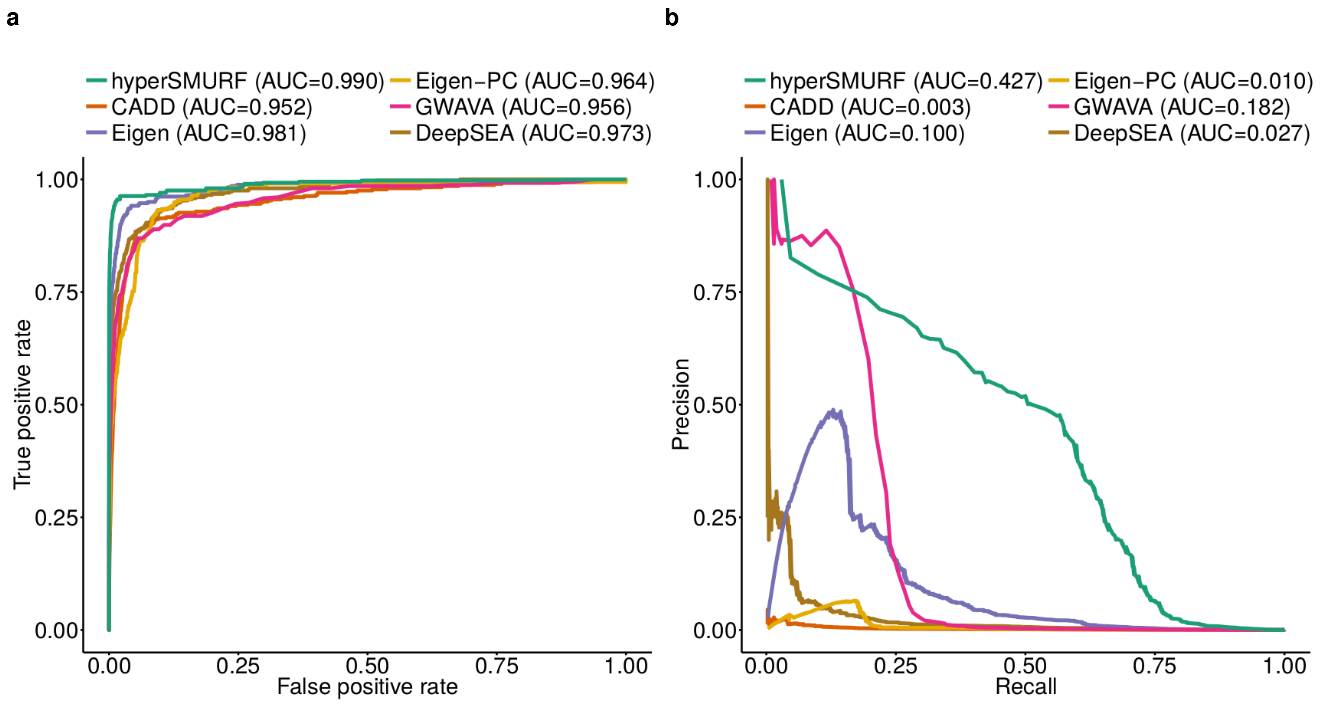

Supplementary Figure 6. Comparison of ROC and PR curves using precomputed scores. a: ROC and **b:** PR curves across methods using precomputed scores with the Mendelian dataset.

a **b** **c**

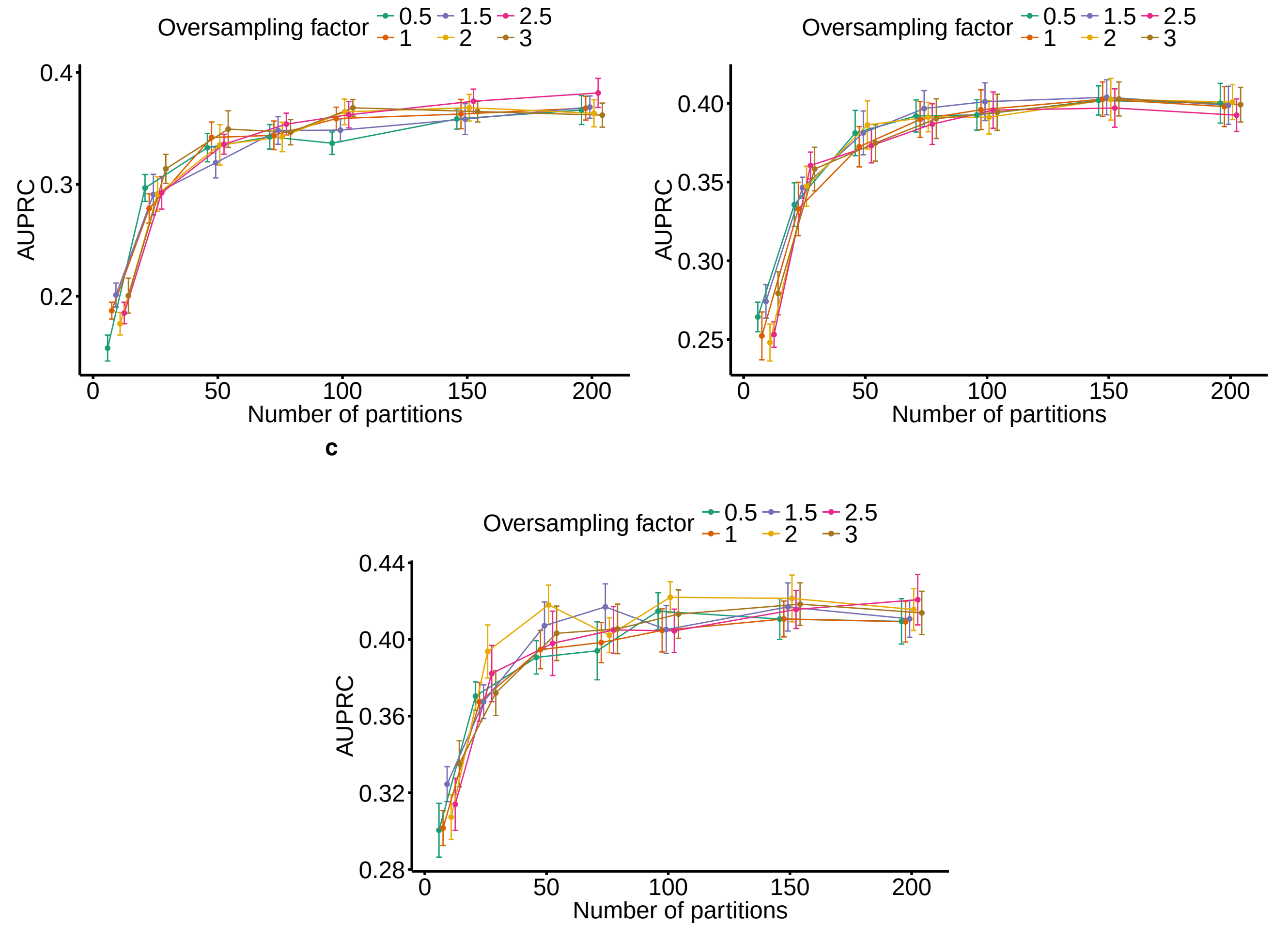

Supplementary Figure 7. ***HyperSMURF*** **parameter tuning with fixed undersampling factor.** Area under the PR curve (AUPRC) as a function of different *HyperSMURF* partition sizes generated by internal nine-fold cytoband-aware cross-validation using Mendelian data. Curves represent different oversampling factors with a fixed undersampling factor m for each figure: **a:** $m = 1$, **b:** $m = 2$ and **c:** $m = 3$. Error bars represent the standard deviation between ten repetitions of internal nine-fold cross-validation using different folds. The undersampling factor is the ratio of negative examples with respect to positives. Negative examples were randomly sampled without replacement from each partition of the data (see Supplementary Note 2 for details).

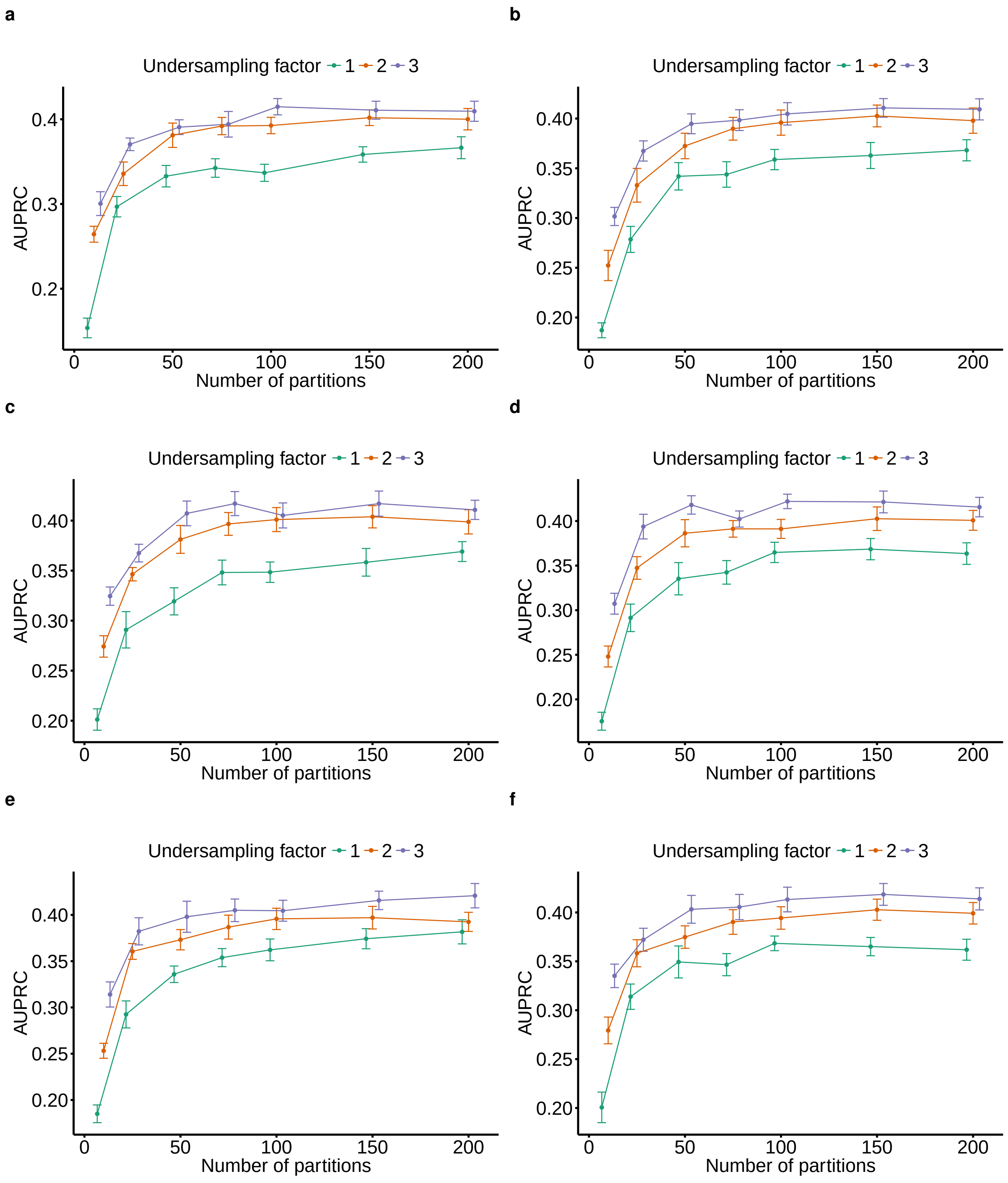

Supplementary Figure 8. ***HyperSMURF* parameter tuning with fixed oversampling factor.** AUPRC as a function of different *hyperSMURF* partition sizes generated by internal nine-fold cytoband-aware cross-validation using Mendelian data. Curves represent different undersampling factors with a fixed oversampling factor f for each figure: **a:**, $f = 0.5$, **b:** $f = 1$, **c:** $f = 1.5$, **d:** $f = 2$, **e:** $f = 2.5$, and **f:** $f = 3$. Error bars represent the standard deviation between ten repetitions of internal nine-fold cross-validation using different folds. The oversampling factor is the ratio of synthetic positive examples generated through the SMOTE algorithm with respect to the available number of positive examples (see Supplementary Note 2 for details).

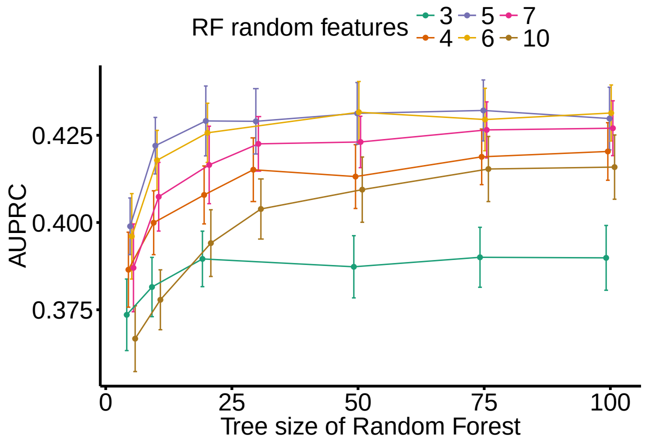

Supplementary Figure 9. Tuning of Random Forest parameters. Area under the PR curve (AUPRC) as a function of different Random Forest sizes (number of decision trees) trained by internal nine-fold cytoband-aware cross-validation. The curves represent the number of random features (3,4,5,6,7,10) used to construct each decision tree of the random forest. The other *hyperSMURF* parameters were set to standard values (Supplementary Table 4). Error bars represent the standard deviation between ten repetitions of internal nine-fold cross-validation using different folds.

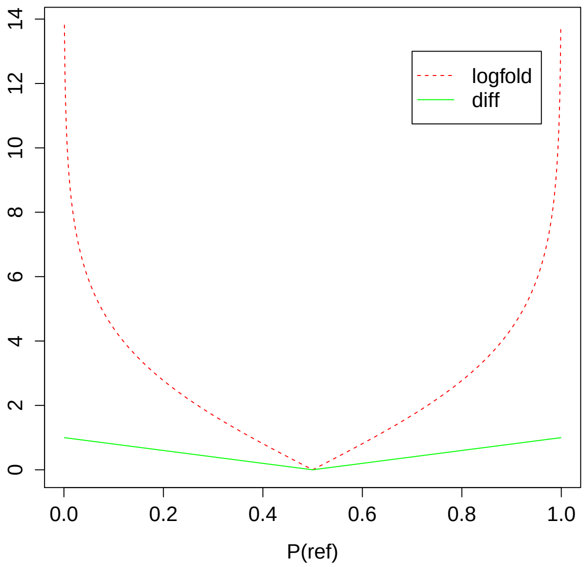

Supplementary Figure 10. Characteristics of the two sets of DeepSEA features. Plot of the functions $logfold$ and $diff$ used to generate the two differents sets of DeepSEA features. $P(reference)$ values between 0 and 1 are represented in abscissa.

Supplementary Note 1: Supplementary Experimental results

In this Supplementary Note we present a thorough analysis of the precision, recall, and F-score as a function of the score predicted by *hyperSMURF* and several state-of-the-art scoring methods. In order to obtain a common basis for the comparison between methods, we rescaled all the scores in the range $[0, 1]$ (indicated as ”threshold” or “normalized score” in Supplementary Fig. 2-5) through a simple linear transformation.

Precision, Recall and F-score results with GWAS data. *HyperSMURF* and *GWAVA* achieve the best F-score results (Supplementary Fig. 2c), due to their better precision performance (Supplementary Fig. 2a), while maintaining good recall performance across most of the range of normalized score thresholds (Supplementary Fig. 2b). *DeepSEA* achieves good results too, even if worse than *hyperSMURF*; this is not surprising since to train all the methods with these data we used features extracted directly from DNA sequence through deep convolutional networks, developed as part of the overall *DeepSEA* method itself[4] (Supplementary Fig. 2a–c).

The other imbalance-unaware ML-based methods achieve relatively low F-score results: *CADD* obtains a very low F-score peak at a normalized score of about 0.20, due to the low but not negligible precision in the normalized score range of $[0.20.0.50]$, while the sensitivity, high only for low values of the normalized scores, dramatically decreases at a score value of about 0.20 (Supplementary Fig. 2a–c). *Eigen* and *Eigen-PC* maintain a high recall only for score thresholds below 0.50, but the precision is very low for the full range of the normalized scores, thus resulting in poor F-scores (Supplementary Fig. 2a–c). It is likely that in this case, having a large set of input features (about 1800, see *GWAS data* Section in the main manuscript), it is difficult for fully unsupervised learning methods such as *Eigen* and *Eigen-PC* to achieve competitive results.

Balanced accuracy results confirm the behavior of the different methods observed with precision, recall and F-score (Supplementary Fig. 2d). Supplementary Figure 3 shows the details of the precision, recall and F-score for the highest values of the normalized score, and suggests possible ”good” thresholds to detect GWAS regulatory hits at least for *hyperSMURF*(a threshold at about 0.85), and for *GWAVA* and *DeepSEA* methods (a threshold slightly larger than 0.85 – Supplementary Fig. 3).

Precision, Recall and F-score results with Mendelian data. With non-coding Mendelian the overall behavior of the algorithms is similar to that observed with GWAS data, even if the higher imbalance between positive and negative examples with respect to GWAS data leads to overall worse results.

With Mendelian data, *hyperSMURF* and *GWAVA* show the highest precision (Supplementary Fig. 4a), especially in the highest range of the normalized score (Supplementary Fig. 5a and -e), while *CADD* shows the highest sensitivity across almost all the considered thresholds (Supplementary Fig. 4b). Nevertheless *CADD* precision is always close to 0 and increases only for normalized scores very close to 1, i.e. when the recall dramatically declines, thus resulting in a F-score very close to 0 for the full range of the normalized scores, except for a small peak (F-score~ 0.1) for normalized scores very close to 1 (Supplementary Fig. 5b). The highest F-score is achieved by *HyperSMURF*, but also *GWAVA* and to some extent *DeepSEA* obtain good results (Supplementary Fig. 4c), especially in the highest range of the normalized scores (Supplementary Fig. 5a, e and f). The best F-scores of *hyperSMURF* and *GWAVA* are due to their higher precision in the highest range of the normalized score, while maintaining a good recall across all the thresholds: the behavior of the two methods is similar, even if *hyperSMURF* precision is larger than *GWAVA*, thus resulting in a better F-score (Supplementary Fig. 5a and e). This analysis also shows that a good threshold for a classifier (i.e. a classifier with a high F-score) is about 0.97 for *hyperSMURF* (Supplementary Fig. 5a, about 0.99 for *GWAVA* (Supplementary Fig. 4e) and about 0.95 for *DeepSEA* (Supplementary Fig. 5f). *Eigen* and *Eigen-PC* performance depends on the peaks of precision respectively at the highest and lowest score thresholds (Supplementary Fig. 4a), and on the opposite trends of the corresponding recall curves (Supplementary Fig. 4b). However, both recall curves of *Eigen* and *Eigen-PC* drop in correspondence with the peaks of precision, thus resulting in relatively low peaks of the F-score (Supplementary Fig. 4c).

The results in terms of the balanced accuracy confirm the better results obtained with imbalance-aware methods (*hyper-SMURF* and *GWAVA)* as compared to imbalance-unaware methods (Supplementary Fig. 4d).

Supplementary Note 2: Experimental analysis and automatic tuning of the parameters of *HyperSMURF*

HyperSMURF learning depends on several parameters including oversampling and undersampling ratios, the size of the hyper-ensemble (number of partitions), as well as on the learning parameters of the Random Forest (RF), that constitute the base learners of the *HyperSMURF* method. We provide default parameters that work well in the imbalanced settings analyzed in this work (Supplementary Table 4). Nevertheless, we also designed an adaptive *hyperSMURF* model able to automatically learn the "best" learning parameters for a specific learning task. To this end we developed an adaptive learning strategy to automatically learn from the data the best parameters, but avoiding overfitting through an internal cytoband-aware validation and selection of the learning parameters.

More precisely, for each round of the 10-fold cytoband-aware cross-validation (CV), we used the nine partitions of the training set to repeatedly run an internal nine-fold cytoband-aware CV using at each iteration different sets of parameters (Supplementary Table 4). Because of computational complexity issues, we split the parameter search into a first step in which we optimize the *hyperSMURF* learning parameters, and into a second step to optimize the RF learning parameters. In the first step, we optimized the number of partitions n, together with the oversampling factor f and the undersampling factor m, while the parameters of the RF were kept fixed using $t = 10$ decision trees and $d = 5$ randomly selected features. In the second step, we optimized the RF learning parameters (i.e. the number of trees of each RF and the number of randomly selected features), while maintaining fixed the optimized *hyperSMURF* learning parameters. The "split" of the optimization procedure is motivated by the need of reducing the combinatorial complexity that rises by the joint optimization of all the parameters. Finally, we trained an "optimal" *hyperSMURF* model using for every 10-fold CV step the best parameter setting in terms of the highest AUPRC obtained with the internal nine-fold CV. We repeated the cytoband-aware CV on Mendelian data 100 times with standard settings (Supplementary Table 4) and with the selected optimal learning parameters using different seeds to get an average performance that is not dependent on randomization issues. The standard *hyperSMURF* achieved an AUPRC of 0.4195 ± 0.0175, while the optimized algorithm an AUPRC of 0.4431 ± 0.0124, and this difference is significant according to the Wilcoxon rank sum test (p-value $< 10^{-6}$).

To provide an idea about the impact of the different learning parameters on the overall *hyperSMURF* performance, Supplementary Figure 7 shows that increasing the number n of partitions, the performance in terms of AUPRC increases, as expected, because a larger space of the negative variants is explored and the larger size of the hyper-ensemble provides more accurate and reliable results. Nevertheless, we have a steep boost until $n = 100$ and for larger number of partitions the improvement is marginal. In terms of runtime this means that we can safely use $n = 100$ to obtain reasonably good performance. Supplementary Figure 8 shows that the best selection of the undersampling factor m within a partition is also important. Indeed the curves corresponding to $m = 3$ lie steadily over the other curves with lower undersampling ratio, showing on the average an AUPRC increment of about 0.05, independently of the oversampling factor used. The oversampling factor f contributes to the overall performance too (Supplementary Fig. 7), even if the introduced improvement is less significant with respect to the undersampling ratio.

Also the learning parameters of the RF, the base learners of *hyperSMURF*, plays a role in the performance of the hyper-ensemble. Indeed Supplementary Figure 9, as expected, shows that an increasing forest size leads to better performances, even if the AUPRC curve reaches a really fast saturation for forest sizes larger than $t = 20$. Therefore there is no need to increase runtime by increasing the forest size. Moreover also the random number d of selected features is an important learning parameter: Supplementary Figure 9 shows that the best curves are those corresponding to $d = 5$ and $d = 6$, and a relatively large decrement in performance can be registered when suboptimal number of features d are selected. These results reveal that with the standard settings (Supplementary Table 4) we obtain good performances on Mendelian data, while keeping the runtime to a minimum.

Finally, considering that *hyperSMURF* is a hyper-ensemble approach with partitioning of the data, oversampling of positive examples, and undersampling of negatives, we performed experiments to study the impact of each of these "components" on the overall performance of the algorithm. Supplementary Table 5 shows the performance of the full *hyperSMURF* algorithm compared with the results obtained without oversampling, and without partitioning (i.e. by removing the hyper-ensemble approach). Results show that each component of the algorithm (and in particular the hyper-ensemble approach) plays a key role to improve the performance of the method. Comparison with a standard RF (that constitutes the base learner of *hyperSMURF*) shows the very large improvements obtained by *hyperSMURF* with respect to standard state-of-art machine learning algorithms.

Supplementary Note 3. The *hyperSMURF* software R package

The R version of *hyperSMURF* is available from the CRAN repository:
https://cran.r-project.org/package=hyperSMURF.

R package installation. Here, we explain the installation on Linux systems, but the procedure is similar for Mac OS and Windows.

You can install the package by downloading the tarball from the CRAN repository, and then by calling R from the shell command line:

```
$ R CMD INSTALL hyperSMURF_1.1.2.tar.gz
```

Alternatively, you can both download and install the package directly from the R command line:

```
> install.packages("hyperSMURF")
```

Main functionalities implemented in the R package. The package implements both the training (function `hyperSMURF.train`) and the testing (function `hyperSMURF.test`), as well as the fully automated CV of the *hyperSMURF* model (`hyperSMURF.cv`).

The parallel version of the training, testing and CV functions are provided as well through the functions `hyperSMURF.train.paralle` `hyperSMURF.test.parallel` and `hyperSMURF.cv.parallel`. These functions can be used with multi-core architectures and can achieve a quasi linear speed-up with respect the number of available cores.

Other functions include a feature selection step to reduce the dimensionality of the data and to select the most informative features for training and testing (`hyperSMURF.corr.cv.parallel`), a modified version of the *hyperSMURF* algorithm that computes the scores by using a tunable cutoff for the decision of each base RF instead of the estimated probability provided by each base RF (`hyperSMURF.test.thresh`). Other ancillary functions provide functionalities for the implementation of the CV (`do.stratified.cv.data`, `do.stratified.cv.data.from.fold`), and to generate synthetic imbalanced data sets (`imbalanced.data.generator`).

A full description of the available R functions is available in the Reference manual included in the package as PDF and HTML.

Simple usage examples using synthetic data. Here we introduce some simple usage examples using the generator of synthetic imbalanced data included in the R package. At first we load the library:

```
> library(hyperSMURF)
```

Then we construct two imbalanced data sets (training and test set) having both 20 "positive" and 2000 "negative" examples with 10 features (dimension of input data equal to 10 – see the Reference manual for details about the synthetic data generator):

```
> train <- imbalanced.data.generator(n.pos=20, n.neg=2000, n.features=10,
                                     n.inf.features=3, sd=0.1, seed=1);
> test <- imbalanced.data.generator(n.pos=20, n.neg=2000, n.features=10,
                                    n.inf.features=3, sd=0.1, seed=2);
```

Then we can train and test the model with the following code:

```
> HSmodel <- hyperSMURF.train(train$data, train$label, n.part = 10, fp = 2, ratio = 3);
> res <- hyperSMURF.test(test$data, HSmodel);
```

Note that we used 10 partitions of the training data (parameter *n.part* that corresponds to the parameter n in the pseudo-code of the algorithm in Supplementary Note 1), a SMOTE oversampling equal to 2 (parameter *fp* corresponding to the f parameter in the pseudo-code), and undersampling ratio equal to 3 (parameter *ratio* corresponding to the parameter m of the modified second line of the *hyperSMURF* algorithm in Supplementary Note 1). In other words the negative examples were partitioned in 10 sets of equal size (200 examples). Then a different RF was trained using: a) the available 20 positive examples, plus the "augmented" 40 synthetic positive examples obtained by SMOTE convex combination of close positive examples, and b) a set of $3 \times 60 = 180$ negative examples randomly extracted from the partition (see Supplementary Note 1). The obtained *hyperSMURF* model (`HSModel`), that includes 10 different RF (one for each partition), is finally tested on the test set. We can easily obtain the confusion matrix:

```
> y <- ifelse(test$labels==1,1,0);
> pred <- ifelse(res>0.5,1,0);
> table(pred,y);
```

```
    y
pred    0    1
   0 1979    1
   1   21   19
```

The accuracy is 0.9891 and the F-score (more informative in this unbalanced context) is 0.6333. Note that with a RF that does not adopt unbalance-aware learning strategies on the same data we obtain significantly worse results in terms of the F-score:

```
> library(randomForest);
> RF <- randomForest(train$data, train$label);
> res <- predict(RF, test$data);
> y <- ifelse(test$labels==1,1,0);
> pred <- ifelse(res==1,1,0);
> table(pred,y);
    y
pred    0    1
   0 2000   16
   1    0    4
```

The accuracy of the RF is high (0.9930), but the F-score is 0.3333, only about half that of *hyperSMURF*. [1]

To perform a 5 fold CV on a given data set we need only 1 line of R code:

```
> res <- hyperSMURF.cv(train$data, train$labels, kk = 5, n.part = 10, fp = 1, ratio = 1);
```

To compute the AUROC and the AUPRC (respectively the area under the ROC curve and the area under the precision/recall curve) we can use the *precrec* package:

```
> library(precrec);
> labels <- ifelse(train$labels==1,1,0);
> digits=4;
> sscurves <- evalmod(scores = res, labels = labels);
> m<-attr(sscurves,"auc",exact=FALSE);
> AUROC <-  round(m[1,"aucs"],digits);
> AUPRC <-  round(m[2,"aucs"],digits);
> cat ("AUROC = ", AUROC, "\n", "AUPRC = ", AUPRC, "\n");
AUROC =  0.9972
AUPRC =  0.8540
```

We can also apply the version of *hyperSMURF* that embeds a feature selection step on the training data to select the features most correlated with the labels:

```
> res <-hyperSMURF.corr.cv.parallel(train$data, train$labels, kk = 5,
              n.part = 10, fp = 1, ratio = 1, mtry=3, n.feature = 6);
> sscurves <- evalmod(scores = res, labels = labels);
> m<-attr(sscurves,"auc",exact=FALSE);
> AUROC <-  round(m[1,"aucs"],digits);
> AUPRC <-  round(m[2,"aucs"],digits);
> cat ("AUROC = ", AUROC, "\n", "AUPRC = ", AUPRC, "\n");
AUROC =  0.9982
AUPRC =  0.9190
```

Usage examples with genetic data. *HyperSMURF* was designed to predict rare genomic variants, when the available examples of such variants are substantially less than "background" examples. This is a typical situation with genetic variants. For instance, we have only a small set of available variants known to be associated with Mendelian diseases in non-coding regions (positive examples) against the sea of background variants, i.e. a ratio of about 1 : 36,000 between positive and negative examples[5].

Here we show how to use *hyperSMURF* to detect these rare features using data sets obtained from the original large set of Mendelian data[5]. To provide usage examples that do not require more than 1 minute of computation time on a modern

[1] Note that the results may vary slightly due to the randomization in the algorithm.

desktop computer, we considered data sets downsampled from the original Mendelian data set described in the "mendelian data" section of the main manuscript (this data set includes more than 14 millions of genetic variants). In particular we constructed Mendelian data sets with a progressive larger imbalance between Mendelian associated mutations and background genetic variants. We start with an artificially balanced data set, and then we consider progressively imbalanced data sets with ratio "positive:negative" varying from 1 : 10, to 1 : 100 and 1 : 1000. These data sets are downloadable as compressed .rda R objects from http://homes.di.unimi.it/valentini/DATA/Mendelian.

The `Mendelian_balanced.rda` file include 3 objects: `m.subset`, that includes the input features of the balanced examples (406 positives and 400 negatives), `labels.subset`, i.e. the corresponding labels, and `folds.subset` a vector with the number of the fold in which each example will be included according to the 10-fold cytoband-aware CV procedure (see Supplementary Note 2). The following lines of code load the data and perform a 10-fold cytoband-aware CV and compute the AUROC and AUPRC:

```
> load("Mendelian_balanced.rda");
> res <- hyperSMURF.cv(m.subset, factor(labels.subset, levels=c(1,0)), kk = 10, n.part = 2,
fp = 0, ratio = 1, k = 5, ntree = 10, mtry = 6,  seed = 1, fold.partition = folds.subset);

> sscurves <- evalmod(scores = res, labels = labels.subset);
> m<-attr(sscurves,"auc",exact=FALSE);
> AUROC <-  round(m[1,"aucs"],digits);
> AUPRC <-  round(m[2,"aucs"],digits);
> cat ("AUROC = ", AUROC, "\n", "AUPRC = ", AUPRC, "\n");
AUROC =  0.9903
AUPRC =  0.9893
```

Then we can perform the same computation using the progressively imbalanced data sets:

```
# Imbalance 1:10. about 400 positives and 4000 negative variants
> load("Mendelian_1:10.rda");

> res <- hyperSMURF.cv(m.subset, factor(labels.subset, levels=c(1,0)), kk = 10, n.part = 5,
fp = 1, ratio = 1, k = 5, ntree = 10, mtry = 6,  seed = 1, fold.partition = folds.subset);

> sscurves <- evalmod(scores = res, labels = labels.subset);
> m<-attr(sscurves,"auc",exact=FALSE);
> AUROC <-  round(m[1,"aucs"],digits);
> AUPRC <-  round(m[2,"aucs"],digits);
> cat ("AUROC = ", AUROC, "\n", "AUPRC = ", AUPRC, "\n");
AUROC =  0.9915
AUPRC =  0.9583

# Imbalance 1:100. about 400 positives and 40000 negative variants
> load("Mendelian_1:100.rda");
> res <- hyperSMURF.cv(m.subset, factor(labels.subset, levels=c(1,0)), kk = 10, n.part = 10,
fp = 2, ratio = 3, k = 5, ntree = 10, mtry = 6,  seed = 1, fold.partition = folds.subset);

> sscurves <- evalmod(scores = res, labels = labels.subset);
> m<-attr(sscurves,"auc",exact=FALSE);
> AUROC <-  round(m[1,"aucs"],digits);
> AUPRC <-  round(m[2,"aucs"],digits);
> cat ("AUROC = ", AUROC, "\n", "AUPRC = ", AUPRC, "\n");
AUROC =  0.9922
AUPRC =  0.9

# Imbalance 1:1000. about 400 positives and 400000 negative variants
load("Mendelian_1:1000.rda");

> res <- hyperSMURF.cv(m.subset, factor(labels.subset, levels=c(1,0)), kk = 10, n.part = 10,
```

```
fp = 2, ratio = 3, k = 5, ntree = 10, mtry = 6,  seed = 1, fold.partition = folds.subset);

> sscurves <- evalmod(scores = res, labels = labels.subset);
> m<-attr(sscurves,"auc",exact=FALSE);
> AUROC <-  round(m[1,"aucs"],digits);
> AUPRC <-  round(m[2,"aucs"],digits);
> cat ("AUROC = ", AUROC, "\n", "AUPRC = ", AUPRC, "\n");
AUROC =  0.9901
AUPRC =  0.7737
```

As we can see, we have a certain decrement of the performances when the imbalance increases. Indeed when we have perfectly balanced data the AUPRC is very close to 1, while by increasing the imbalance we have a progressive decrement of the AUPRC to 0.9583, 0.9000, till to 0.7737 when we have a 1 : 1000 imbalance ratio. Nevertheless this decline in performance is relatively small compared to that of state-of-the-art imbalance-unaware learning methods (see Fig. 5 in the main manuscript).

We can perform the same task using parallel computation. For instance, by using 4 cores with an Intel i7-2670QM CPU, 2.20GHz, less than 1 minute is necessary to perform a full 10-fold cytoband-aware CV using 406 genetic variants known to be associated with Mendelian diseases and 400,000 background variants:

```
res <- hyperSMURF.cv.parallel(m.subset, factor(labels.subset, levels=c(1,0)),
                  kk = 10, n.part = 10, fp = 2, ratio = 3, k = 5, ntree = 10,
                  mtry = 6,  seed = 1, fold.partition = folds.subset, ncores=4);
```

Of course the training and CV functions allow to set also the parameters of the RF ensembles, that constitute the base learners of the *hyperSMURF* hyper-ensemble, such as the number of decision trees to be used for each RF (parameter `ntree`) or the number of features to be randomly selected from the set of available input features at each step of the inductive learning of the decision tree (parameter `mtry`). The full description of all the parameters and the output of each function is available in the PDF and HTML documentation included in the *hyperSMURF* R package.

References

1. Eilbeck, K. *et al.* The sequence ontology: a tool for the unification of genome annotations. *Genome Biol* **6**, R44 (2005). URL http://dx.doi.org/10.1186/gb-2005-6-5-r44.
2. Jäger, M. *et al.* Jannovar: a java library for exome annotation. *Human mutation* **35**, 548–55 (2014). URL http://www.ncbi.nlm.nih.gov/pubmed/24677618.
3. Pruitt, K. D. *et al.* RefSeq: an update on mammalian reference sequences. *Nucleic acids research* **42**, D756–63 (2014). URL http://nar.oxfordjournals.org/content/42/D1/D756.long.
4. Zhou, J. & Troyanskaya, O. G. Predicting effects of noncoding variants with deep learning-based sequence model. *Nature methods* **12**, 931–934 (2015). URL http://dx.doi.org/10.1038/nmeth.3547.
5. Smedley, D. *et al.* A Whole-Genome Analysis Framework for Effective Identification of Pathogenic Regulatory Variants in Mendelian Disease. *The American Journal of Human Genetics* **99**, 595–606 (2016). URL http://linkinghub.elsevier.com/retrieve/pii/S0002929716302786.
